# Cell‐Type‐Specific Autophagy in Human Leukocytes

**DOI:** 10.1096/fj.202402377R

**Published:** 2025-06-12

**Authors:** Linh V. P. Dang, Alexis Martin, Julian M. Carosi, Jemima Gore, Sanjna Singh, Timothy J. Sargeant

**Affiliations:** ^1^ Lysosomal Health in Ageing, Lifelong Health South Australian Health and Medical Research Institute (SAHMRI) Adelaide South Australia Australia; ^2^ Adelaide Medical School The University of Adelaide Adelaide South Australia Australia; ^3^ School of Biological Sciences, Faculty of Sciences, Engineering and Technology The University of Adelaide Adelaide South Australia Australia; ^4^ SAHMRI Clinical Trials Platform (CTP) South Australian Health and Medical Research Institute (SAHMRI) Adelaide South Australia Australia

**Keywords:** aging, autophagic flux, autophagy, blood, human, sex

## Abstract

Autophagy is a naturally conserved mechanism crucial for degrading and recycling damaged organelles and proteins to support cell survival. This process slows biological aging and age‐related disease in preclinical models. However, there has been little translation of autophagy to the clinic, and we have identified a lack of measurement tools for physiological human autophagy as a barrier. To address this, we have previously developed a direct measurement tool for autophagy in pooled human peripheral blood mononuclear cells (PBMCs) in the context of whole blood. In order to better understand how autophagy behaves and changes in humans, we measured human autophagic flux using flow cytometry in 19 cell subpopulations in whole blood to retain physiological flux. Autophagic flux was different between different cell types, being different within different monocyte, B lymphocyte, natural killer cell, and T lymphocyte subtypes. Autophagic flux also varied with sex, being higher in monocytes in females compared with males. In keeping with previous observations in humans, autophagy also increased with aging at subpopulation levels. Importantly, we found that only monocytes—specifically, nonclassical monocytes—displayed robust increased autophagic flux following amino acid withdrawal, underscoring the importance of population selection for measurement of autophagic flux during nutrient restriction studies in humans. Collectively, these data show PBMC population‐level analysis improves sensitivity of human autophagic flux measurement.

## Graphical abstract

We previously established a whole‐blood assay to measure autophagic flux by adding chloroquine to whole blood to inhibit lysosomal degradation of autophagosomes. We adapted this technique to flow cytometry to quantify cell‐type‐specific autophagy. We found monocytes are nutrient responsive to amino acids, while lymphocytes were not. Sex differences were observed in select cell subsets. Importantly, autophagy in whole blood was higher than in standard culture conditions, underscoring the value of physiological context. Figure was made using Biorender.com.

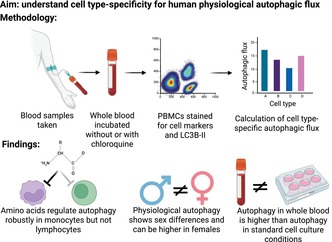

**Abbreviations**
aa: amino acid
BafA: bafilomycin A
BSA: bovine serum albumin
CM: central memory
CQ: chloroquine
DPBS: dulbecco's phosphate‐buffered saline
ELISA: enzyme‐linked immunosorbent assay
EM: effector memory
FBS: fetal bovine serum
IL: interleukin
KO: knockout
MFI: geometric Mean Fluorescence Intensity
NK: natural killer
PBMC: peripheral blood mononuclear cell
RT: room temperature
TEMRA: effector memory cells reexpressing CD45RA
Treg: regulatory T cell
WB: whole blood

## 1 Introduction

Macroautophagy (hereafter referred to as autophagy) is a nutrient‐ and stress‐responsive process that supports cellular resilience by recycling intracellular material. This allows the provision of nutrients during starvation, clearance of invading pathogens, suppression of inflammation, and maintenance of the mitochondrial network [1, 2, 3, 4]. The consequences of poorly functioning autophagy in mouse models include accelerated biological aging [4, 5, 6] and age‐related diseases that include atherosclerosis, dementia, and different cancers [7]. Autophagy is also modifiable in cell and preclinical models using pharmacological and nutrition‐based interventions [8, 9]. This means that autophagy has huge potential as a pathway that could slow or delay the onset of age‐related disease.

Although autophagy has immense translational potential, autophagy‐based research generally has not progressed beyond preclinical models, although there have been rare exceptions [10, 11, 12]. The reason for this is in part because autophagy is very difficult to measure in people [13]. To address this block to translation, we recently developed a method of measuring autophagic flux in peripheral blood mononuclear cells (PBMCs) by treating whole blood with the lysosome inhibitor chloroquine (CQ). PBMCs are then isolated, and microtubule associated protein 1 light chain 3 beta (MAP1LC3B) isoform II/LC3B‐II is measured to derive autophagic flux [14], a dynamic process that analyzes the rate at which autophagy captures and degrades intracellular substrates in the lysosome. Maintaining PBMCs in whole blood during blockade of lysosomal degradation is important because it leaves intact physiological concentrations of nutrients and hormones such as insulin.

PBMCs comprise a diverse group of immune cells, including T lymphocytes, B lymphocytes, natural killer (NK) cells, and monocytes, each with distinct functions in immune surveillance, pathogen clearance, and tissue homeostasis. These subsets exhibit characteristic changes with aging and chronic disease, contributing to immune senescence and altered inflammatory responses (Table S1). For example, the expansion of memory T‐cell populations, the decline in naïve lymphocytes [15, 16, 17], and the shift in monocyte population composition are changes that occur with aging [18]. Understanding how autophagic flux varies among these subsets under different conditions is critical; this is important as autophagy plays a central role in regulating immune cell function and metabolism [19], and this will lead to tools that are useful for the measurement of physiological autophagic flux in humans.

In order to develop more precise measures of autophagy in humans that are robust to changes in proportions of cellular populations in the PBMC pool, we need to understand what autophagy looks like in specific cellular subpopulations. This is especially important as development and aging [15, 20, 21], obesity and weight loss [22], exercise [23], high‐fat food [24] and diseases [25] can all impact cell characteristics and population fractions within the PBMC pool. We aimed to address this issue by developing a physiological autophagic flux measurement using flow cytometry in young and middle‐aged adults to evaluate autophagic flux in 19 different cell subpopulations, including T cells, B cells, NK cells, and monocytes (detailed in Supplementary Table S1). Understanding these differences will allow for correct interpretation of autophagy measurements in human blood in future clinical trials, and the development of recommendations for targeting PBMC subpopulations in a study‐appropriate manner.

## 2 Results

### 2.1 Physiological Autophagic Flux Retained in Whole Blood but Not in RPMI or Plasma

LC3B‐II flux was defined as the difference in LC3B‐II abundance with the lysosomal inhibitor chloroquine (CQ) minus the LC3B‐II abundance in a parallel sample where CQ was not added. While autophagic flux in PBMCs cultured in whole blood or culture media (RPMI) has been successfully measured using western blot and enzyme‐linked immunosorbent assay (ELISA) [12, 26], and LC3B levels have been analyzed in various leukocyte populations using flow cytometry [12, 26, 27, 28], physiological autophagic flux in whole blood has not been measured in different cell types using flow cytometry. We, therefore, aimed to do this by culturing whole blood without and with CQ and analyzing autophagy in cell types using flow cytometry for LC3B‐II. To evaluate antibody specificity for measuring LC3B‐II flux, we analyzed wild‐type and LC3B knockout (KO) HEK 293 T cells. We observed a higher signal for LC3B‐II in CQ‐treated samples compared to control samples, but only in wild‐type HEK cells—not in the LC3B KO cells (Figure S1A,B). Similarly, LC3B‐II flux was observed in PBMCs (Figure S1) where LC3B staining was appreciably higher than the IgG isotype control.

We subsequently investigated physiological LC3B‐II flux (measured in the context of whole blood) of major blood cell types by flow cytometry, including monocytes (classical, intermediate and nonclassical monocytes), T lymphocytes (CD4 T lymphocytes: T regulatory cells (Treg), naïve, central memory (CM), effector memory (EM), and terminally differentiated (TEMRA), and CD8 T lymphocytes: naïve, CM, EM and TEMRA), B lymphocytes (naïve and memory B cells), and natural killer (NK) cells (CD56^hi^C16^−^, CD56^hi^CD16^+^, CD56^dim^CD16^−^, and CD56^dim^CD16^+^ NK cells) [15, 29] (gating strategy is shown in Figure S1D–U).

**FIGURE 1:**
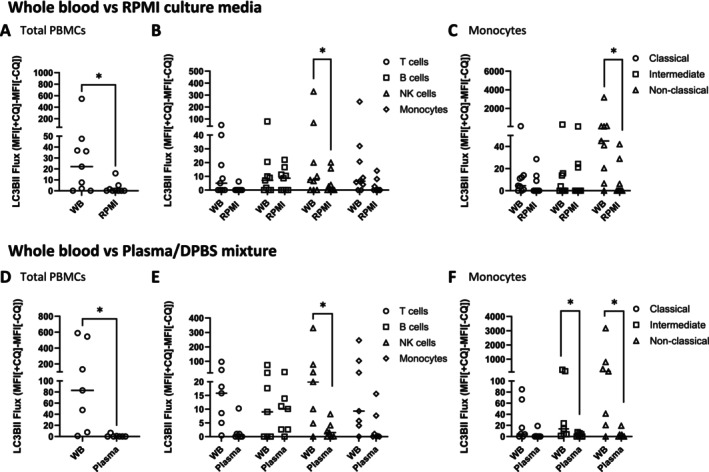
Autophagic flux measurement in physiological and nonphysiological environments. LC3B‐II flux in PBMCs exposed to CQ in whole blood compared to RPMI containing 10% FBS (*N* = 9) is shown as follows: Total PBMCs (A) T cells, B cells, NK cells, monocytes (B), Monocyte subpopulations: classical, intermediate, nonclassical monocytes (C). LC3B‐II flux in PBMCs exposed to CQ in whole blood compared to 1:1 cognate plasma:DPBS (*N* = 7) is shown as follows: Total LC3B‐II flux (D), T cells, B cells, NK cells, monocytes (E), Monocyte subpopulations: Classical, intermediate, nonclassical monocytes (F). Wilcoxon matched‐paired signed rank tests were used. *p < 0.05. Bars = median. Datapoints = participants.

LC3B‐II flux was measured in different environments including the addition of lysosomal inhibitors to whole blood, nutrient‐rich artificial media (RPMI containing 10% FBS, a common culture medium for PBMCs) or in diluted cognate plasma/Dulbecco's phosphate‐buffered saline (DPBS) mixed in a ratio of 1:1 (Figure 1A–F, S2A–J). We observed a significant reduction in overall LC3B‐II flux in PBMCs cultured in RPMI containing 10% FBS (Figure 1A–C) or plasma/DPBS (Figure 1D–F) compared to PBMCs exposed to CQ in the context of whole blood. This was particularly evident in NK cells (Figure 1B,E), nonclassical monocytes in RPMI medium containing 10% FBS (Figure 1C), and intermediate and nonclassical monocytes in cognate plasma/DPBS (Figure 1F).

### 2.2 Basal Autophagic Flux Is Intrinsically Different in Different Subpopulations of the PBMC Pool

Assessing physiological autophagy at the level of leukocyte subpopulations is important to understand how autophagic flux in the PBMC pool as a whole may be impacted by shifting subpopulation fractions, which is known to occur with factors such as aging [15]. To understand what cell‐type‐specific physiological autophagic flux looks like, we analyzed LC3B‐II flux in whole blood in 43 participants. Participant characteristics are presented in Table S2.

**FIGURE 2:**
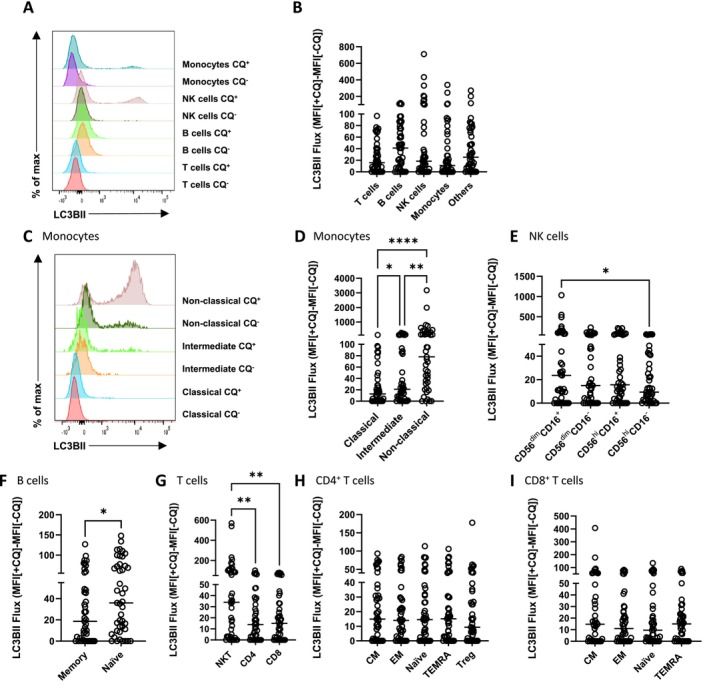
Basal physiological autophagic flux is intrinsically different in different cell types. Flow cytometry histogram representing LC3B‐II fluorescent signal in whole blood treated with or without CQ (A). Summary data of LC3B‐II flux in different cell populations, including T cells, B cells, NK cells, monocytes, and others (B). Histogram of LC3B‐II levels of monocyte subpopulations in whole blood treated with or without CQ including classical, intermediate, and nonclassical monocytes (C). Summary data of LC3B‐II flux in different subpopulations including Monocyte (D), NK cells (E), B cells (F), T cells (G), CD4 T cells (H), CD8 T cells (I). Measurement of LC3B‐II flux was performed in *N* = 43 people. Friedman test with multiple comparison and Wilcoxon matched‐paired signed rank test analysis were used. *p < 0.05, **p < 0.01, ***p < 0.001). Bars = median. Datapoints = participants.

Among broad cell type classifications, no significant differences in LC3B‐II flux were observed (Figure 2A,B). Within each broad cell population, nonclassical monocytes had the highest LC3B‐II flux of the monocytes (Figure 2C,D); CD56^dim^CD16^+^ cells had the highest flux within the NK cells (Figure 2E); naive B cells exhibited significantly higher LC3B‐II flux compared to memory B cells (Figure 2F); and higher LC3B‐II flux was found in NKT cells compared to CD4 and CD8 T cells (Figure 2G). Granulocytes showed very high LC3B‐II flux (Figure S3A). The proportion of flux contributed by each cell type to the overall flux of the PBMC pool varied significantly from person to person, both in absolute (Figure S3B) and relative amounts (Figure S3C).

### 2.3 Basal Autophagic Flux in Different Cell Types Is Highly Correlated

**FIGURE 3:**
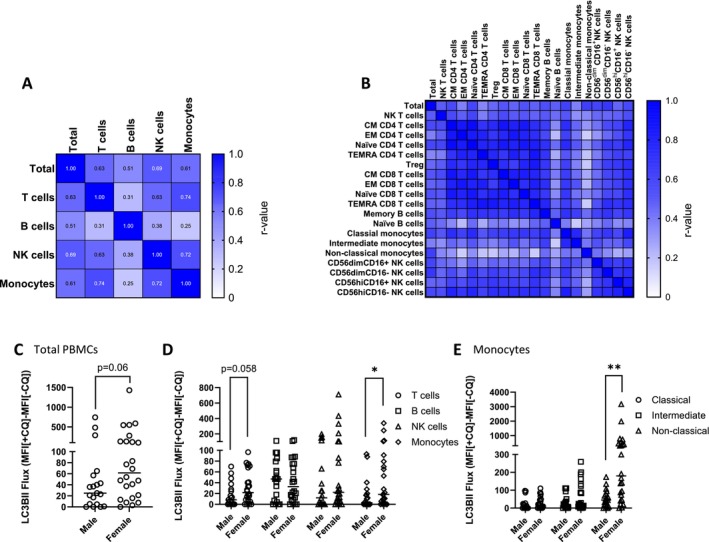
Autophagic flux in different cell types is correlated and shows sex‐related differences. A heat map showing Spearman r value for the correlation between total PBMC LC3B‐II flux and LC3B‐II flux of individual cell populations including T cells, B cells, NK cells and monocytes (A) and flux of individual cell subpopulations (B). Graph showing the difference in LC3B‐II flux between male and female in total PBMCs (C) and PBMC populations: T cells, B cells, NK cells and monocytes (D) and monocyte subpopulations (E). Mann–Whitney tests were used. *p < 0.05, **p < 0.01. Bars = median. Datapoints = participants.

While we observed differences in basal LC3B‐II flux across various subpopulations, we next investigated whether the flux of each individual cell type correlates with the total PBMC flux. The correlation matrix showed a statistically significant correlation between the flux of the total PBMCs and individual cell populations (T cells, B cells, NK cells, and monocytes) (Figure 3A and Table S3A) as well as subpopulations (Figure 3B and Table S3H). Correlation was also statistically significant among each cell type (T cells, B cells, NK cells, and monocytes) and their corresponding subpopulations (Figure S3D–I, Table S3B–G). The results suggest that basal LC3B‐II flux of individual cell populations is highly correlated with the total flux of the whole blood under physiological conditions.

Sex differences in autophagy have been reported in various animal studies [30, 31, 32]. We also analyzed the basal LC3B‐II flux with regard to sex and observed a trending difference in total LC3B‐II flux between males and females (Figure 3C (*p* = 0.06) and Table S4). However, when examining individual cell populations, we noted that females had significantly higher LC3B‐II flux compared to males in monocytes (Figure 3D, Table S4), particularly in nonclassical monocytes (Figure 3E, Table S4).

### 2.4 Basal Autophagic Flux Positively Correlates With Age in Young Adults

**FIGURE 4:**
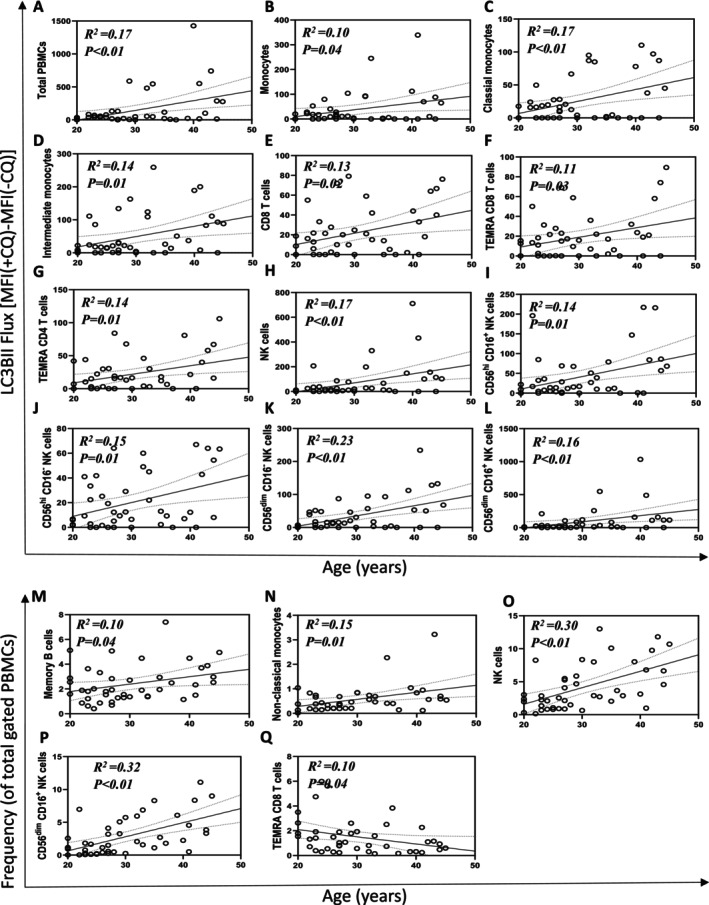
Autophagic flux increases with human age at the cell‐type level. Linear regression between age and LC3B‐II flux (r^2^ and *p*‐value are displayed on graphs) of total PBMCs (A); Monocytes (B), classical monocytes (C), intermediate monocytes (D); CD8 T cells (E), TEMRA CD8 T cells (F) and TEMRA CD4 T cells (G); NK cells (H) CD56^hi^CD16^+^ NK cells (I), CD56^hi^CD16^−^ NK cells (J), CD56^dim^CD16^−^ NK cells (K), and CD56^dim^CD16^+^ NK cells (L). Linear regression between age and proportions of cell populations of total PBMC pool including memory B cells (M), nonclassical monocytes (N), NK cells (O), CD56^dim^CD16^+^ NK cells (P) and TEMRA CD8 T cells (Q). Datapoints = participants.

As age has been reported to be positively correlated with autophagic flux in the PBMC pool in mid to late adulthood (35–70 years of age) in people at risk of type‐2 diabetes [12], we sought to investigate whether this is also true for healthy younger individuals. Crucially, we also wanted to determine whether PBMC subpopulations also exhibited increases in autophagy with age in younger adults. We observed a positive correlation between age (in young adults) and LC3B‐II flux in total PBMCs (Figure 4A); and other populations including monocytes (Figure 4B) and related subpopulations—classical monocytes (Figure 4C), intermediate monocytes (Figure 4D); T‐cell subpopulations including CD8, TEMRA CD8, and TEMRA CD4 T cells (Figure 4E–G); NK cells (Figure 4H) and all subpopulations (Figure 4I–L). Nonclassical monocytes trended with a *p*‐value of 0.053, *R* ^2^ = 0.09 (data not shown). However, after normalizing for sex, autophagic flux of monocytes (*p* = 0.07, *R* ^2^ = 0.15) and classical monocytes (*p* = 0.89, *R* ^2^ = 0.15) lost significance. However, other significant correlations remained including total PBMCs (*p* = 0.01, *R* ^2^ = 0.18), NK cells (*p* = 0.01, *R* ^2^ = 0.18); and T‐cell subpopulations (TEMRA CD4 T cells (*p* = 0.02, R^2^ = 0.14), CD8 T cells (*p* = 0.03, *R* ^2^ = 0.15), TEMRA CD8 T cells (*p* = 0.03, *R* ^2^ = 0.11)), intermediate monocytes (*p* = 0.03, *R* ^2^ = 0.16), NK cell subpopulations (CD56^dim^CD16^+^ NK cells (*p* = 0.02, *R* ^2^ = 0,15), CD56^dim^CD16^−^ NK cells (*p* < 0.01, *R* ^2^ = 0.23), CD56^hi^CD16^+^ NK cells (*p* < 0.01, *R* ^2^ = 0.19), and CD56^hi^CD16^−^ NK cells (*p* = 0.01, *R* ^2^ = 0.15)).

Certain cell populations are reported to change with age with regards to abundance [15], therefore we performed regression analyses and found a significant positive correlation of age in young adults with the frequency of memory B cells (Figure 4M), nonclassical monocytes (Figure 4N), NK cells (Figure 4O) and CD56^dim^CD16^+^ NK cells (Figure 4P), while TEMRA CD8 T cells showed a negative correlation (Figure 4Q). After normalizing for sex in the analysis, we found only NK cells (*p* < 0.01, *R* ^2^ = 0.31), TEMRA CD8 T cells (*p* = 0.02, *R* ^2^ = 0.14), nonclassical monocytes (*p* = 0.01, *R* ^2^ = 0.16), and CD56^dim^CD16^+^ NK cells (*p* < 0.01, *R* ^2^ = 0.32) retained their significant correlation.

To investigate whether the correlation between basal autophagic flux and age in young adults is driven by elevated inflammatory cytokine abundance (interleukin‐1 (IL‐1) and interleukin‐6 (IL‐6) have been shown to increase with age [33]), we analyzed IL‐1 and IL‐6 in participant plasma. We did not observe any correlation between IL‐1 or IL‐6 and age (Figure S4A&B), nor with autophagic flux of total PBMCs (Figure S4C&O). Similarly, no correlation was found between autophagic flux and cytokine abundance in cell subpopulations where autophagic flux was shown to correlate positively with aging in young adults (monocytes (classical and intermediate monocytes, Figure S4D–F & S4P–R), T‐cell subpopulations (Figure S4G–I & S4S–U), and NK cells and their subpopulations (Figure S4J–N & S4V–Z)).

### 2.5 Autophagy Induced by Nutrient Restriction Shows Cell‐Type Specificity

Autophagic flux is activated by nutrient restriction, because nutrients stimulate mTORC1, which inhibits the initiation of autophagy [34]. We wanted to determine whether sensitivity to nutrient restriction was cell‐type‐specific, as this has implications for how human autophagy is monitored during nutritional intervention studies.

**FIGURE 5:**
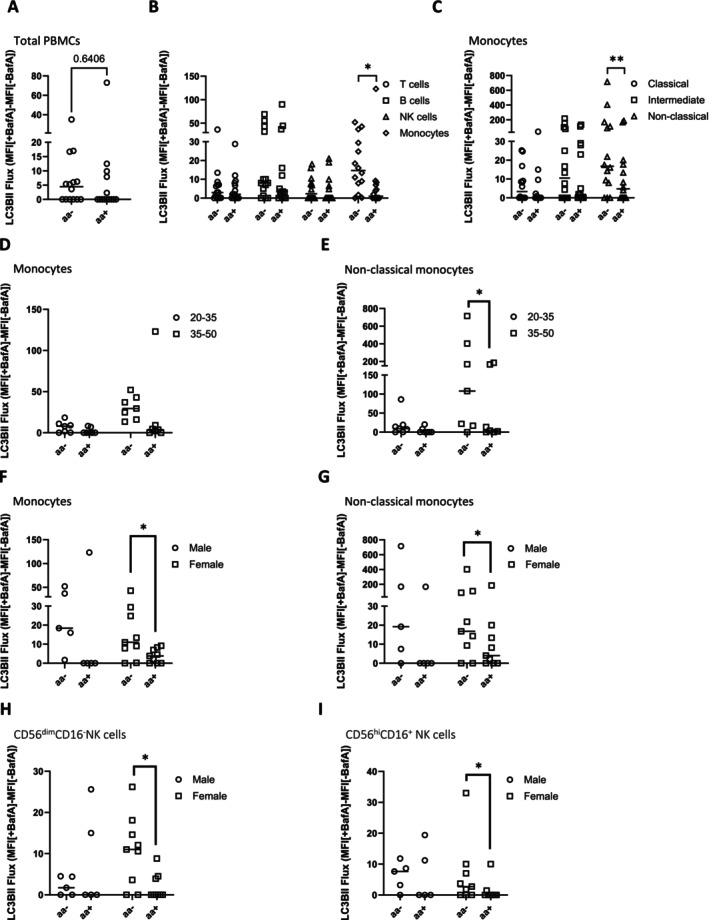
Analysis of LC3B‐II flux of isolated PBMCs cultured under amino acid restriction. LC3B‐II flux was measured in isolated PBMCs cultured in amino acid‐free RPMI containing 10% dFBS (aa^−^) or the same medium spiked with amino acids (aa^+^) with or without BafA (*N* = 14) as follows: Total PBMCs (A), cell populations: T cells, B cells, NK cells, and monocytes (B) monocyte subpopulations (C). These differences were further stratified into age group [20‐35 and 35‐50] and presented as LC3B‐II flux of monocytes (D) and nonclassical monocytes (E). Statistical differences in LC3B‐II between aa^−^ and aa^+^ were also analyzed with respect to sex for monocytes (F), nonclassical monocytes (G), CD56^dim^CD16^−^ (H) and CD56^hi^CD16^+^NK cells (I). Wilcoxon matched‐paired signed rank test was used (*p < 0.05, **p < 0.01). Bars = median. Datapoints = participants.

We cultured PBMCs in RPMI formulations with amino acids (aa^+^) and without amino acids (aa^−^), both containing 10% dialyzed fetal bovine serum (dFBS) for 1 h at 37°C and observed no change in total LC3B‐II flux (Figure 5A). However, significant increases in LC3B‐II flux were observed in monocytes (Figure 5B) and nonclassical monocytes (Figure 5C). Glucose did not elicit a strong response (Figure S5A,B) during the incubation period. When data for autophagic flux were categorized into groups of 20–35 or 35–50 years of age, LC3B‐II flux of monocytes showed no significant difference between the aa^−^ and aa^+^ conditions (Figure 5D). However, significantly higher autophagic flux in response to amino acid restriction was observed in nonclassical monocytes in the 35–50‐year‐old group (Figure 5E), but not in the 20–35‐year‐old group. Significantly higher LC3B‐II flux was observed only in females for both monocytes (Figure 5F) and nonclassical monocytes (Figure 5G) in aa^−^ compared to the aa^+^ condition. Lack of significance in males was likely due to a lack of statistical power. Surprisingly, even though total NK cells were not significantly different with regard to amino acid restriction, we observed increased autophagic flux in CD56^dim^CD16^−^ (Figure 5H) and CD56^hi^CD16^+^ NK cells (Figure 5I) as a result of aa withdrawal in females.

## 3 Discussion

In the current study, we confirmed the importance of using whole blood to measure physiological autophagic flux. We further found that autophagic flux showed cell‐type‐specific variation, and that a positive correlation between age and autophagic flux exists at the total PBMC pool and individual cell‐type levels. Sex also impacted autophagic flux. Additionally, we identified monocytes, particularly nonclassical monocytes, as the most nutrient‐sensitive cells, making them well‐suited for nutritional intervention studies. This study employed flow cytometry to measure autophagy levels in different PBMC subpopulations. While using western blot and ELISA has been used to measure autophagic flux in PBMCs with reasonable accuracy [12, 26], flow cytometry offers the advantage of assessing the autophagic flux of individual populations [35], which is beneficial in the context of aging and disease. In this study, we combined the ability of flow cytometry to monitor cell subpopulations with the physiological relevance of methodology we developed whereby we add a lysosomal inhibitor (CQ) to whole blood, thus maintaining an intact physiological environment. Together, these observations provide useful guidance on how to monitor human autophagy in a study‐appropriate manner.

Consistent with previous research [12], we observed a positive correlation between age and basal autophagic flux. While this had previously been reported in individuals aged 35–75 who were on average obese [12], the current study is the first to report a positive correlation between age and autophagy in a young to middle‐aged adult cohort, and importantly at the leukocyte subpopulation level. We found a positive correlation between age and autophagic flux in monocytes, NK cells, and T lymphocytes and their subpopulations. Although we are the first to see a positive correlation between human autophagy and age in leukocyte subpopulations, a positive correlation has been noted between autophagy and age in mouse PBMCs and mouse adipose tissue [32, 36], and several studies also report increasing mitophagy with age in animal models [37]. These observations force us to reconsider the widely held consensus that autophagy universally declines with age [6].

Other human studies that have described how autophagy changes with age used methodology that left conclusions open to interpretation. For example, LC3B‐II levels are reduced in CD4 T cells from older individuals compared to young counterparts [38], and increased numbers of autophagosomes are present in CD4 T cells in older individuals, along with inconsistent changes in autophagy‐related gene expression [39]. However, these studies have not documented autophagic flux or have performed experiments under nonphysiological conditions. The relevance of these findings to our work is therefore difficult to ascertain.

The increased autophagy that we have observed (this study and Bensalem et al. [12]) could result from a homeostatic response aimed at coping with the molecular damage that increases with aging [40]. This was, however, cell‐specific, suggesting that specific cell types may either accumulate more molecular damage or have a greater ability to increase autophagy to cope with this damage compared to others. Autophagy is known to increase in response to cellular damage—for example, mitochondrial depolarization and lysosomal permeabilization both recruit signaling pathways that result in increased mitophagy or lysophagy, respectively [3, 41]. Curiously, and in line with what we have observed with autophagy in the current study, adaptive proteostatic stress responses have previously been observed in NKT cells and T lymphocytes; both upregulate HSP70 in response to aging [16]. Collectively, our data and these studies suggest that autophagic flux may increase with age in PBMC cell types described herein, and that, in these cell types, autophagy is upregulated in response to age‐related challenges.

We observed sex‐dependent changes in autophagy in the current study where females had more autophagy than males. Other research groups have observed sex‐related differences in autophagy (or autophagy‐related readouts such as P62 abundance and lysotracker staining) in animal models [42, 43] and the sex hormone estrogen by itself is sufficient to change autophagy [44, 45]. We have previously observed sex‐dependent increases in autophagy in females: in a separate human cohort [46], and have also identified sex differences in response to aging and high‐fat diet feeding in mice [32]. It is noteworthy that enhanced autophagy has been linked to increased lifespan in mice [47]. Although speculative, the idea that increased autophagy could be at least partially responsible for the general observation that women live longer than men under nearly any circumstance [48] and tend to age more slowly than men [49, 50] deserves further research.

Nutritional withdrawal has been shown to induce autophagy through different mechanisms, including the activation of AMPK and suppression of mTORC1 activity, and subsequent activation of ULK1, a key initiator of autophagy (reviewed in [51]). In the current study, we observed population‐specific changes in autophagic flux in response to nutrient restriction. While lymphocyte flux remained relatively stable in different ex vivo conditions, monocytes (especially nonclassical monocytes) exhibited significant responses to amino acid restriction. Consistent with this observation, monocytes are more sensitive than lymphocytes to fasting and exercise with regard to the steady‐state abundance of autophagy proteins [52]; autophagic flux, however, has not previously been assessed. Zhang and colleagues also noted monocytes increase autophagy in response to starvation [53]. Although it has been shown that monocytes are nutrient responsive, T lymphocyte autophagy is also likely responsive to different stimuli not tested in the current study; T lymphocytes increase autophagy in response to growth‐stimulating factors anti‐CD3 antibody and IL‐2 [35, 54].

The ability to induce autophagy for nutrient recovery in response to starvation promotes survival under nutrient stress [55, 56]. However, this is not the immune system's only coping mechanism with regards to nutrient restriction. T lymphocytes [57] and monocytes [58, 59] can relocate from the periphery to the nutrient‐rich bone marrow during starvation, which has been suggested as a mechanism to protect cells during nutrient restriction. Jordan and colleagues noted the profound migration of Ly‐6C^hi^ monocytes (equivalent to classical monocytes in humans) from blood to bone marrow, while the numbers of Ly‐6C^lo^ (equivalent to nonclassical monocytes in humans) did not differ significantly in mice after 4 h of starvation [59]. Nonclassical monocytes, which can more effectively induce autophagy upon starvation, might survive better in the periphery during nutrient depletion compared to other cell populations that may need to migrate to a nutrient‐rich environment such as the bone marrow. The changes in cell populations in peripheral blood during starvation suggest the importance of using individual autophagic flux to better monitor changes in autophagy activity. Given their responsiveness to nutrients, monitoring autophagy in monocytes—especially nonclassical monocytes—rather than B or T lymphocytes could provide a more sensitive approach to determining whether nutrient restriction promotes autophagy in humans.

The study has several limitations. We used CQ as a lysosomal inhibitor to measure autophagic flux, and this may induce noncanonical autophagy [60]. The contribution of this to our measurements is currently unknown—the development of useful biomarkers of noncanonical autophagy would help to clarify such contributions. While BafA has been shown not to induce noncanonical autophagy [60], its vehicle, DMSO, has been shown to induce hemolysis in whole blood [26], making measurement of autophagy in whole blood using BafA open to artifact. Moreover, PBMCs treated with BafA exhibited comparable autophagic flux to those treated with CQ [26]; noncanonical autophagy may not be a dominant component of our CQ‐based measurement of autophagic flux in PBMCs.

Additionally, our analysis was limited to LC3B as a marker of autophagy; PBMCs may express and use other ATG8 family proteins, which could confound interpretation. The use of LC3B‐II flux measurements in a cross‐sectional design (rather than longitudinal) also limits our ability to infer age‐related changes in autophagic flux. Moreover, restricting participant recruitment to an Australian cohort may introduce geographic or population‐specific bias.

In conclusion, results from this study indicate how best to measure autophagy in human research. We have demonstrated that human cells maintained in artificial media could be subject to large changes in autophagy, meaning that autophagy observed in culture does not resemble autophagy that takes place within a human being. This provides evidence supporting the use of whole blood to assess physiological autophagic flux. We have also shown that autophagy is different in different cellular populations. Thus, changing subcellular population fractions within the PBMC pool could impact the interpretation of human autophagy studies that measure the whole PBMC pool. We further demonstrated that autophagic flux in humans varies with aging and sex, even at the cell subpopulation level. This shows that both factors must be considered when designing studies for the measurement of autophagy in humans. We also show that autophagy in certain cell types (specifically monocytes/nonclassical monocytes) is more sensitive to nutrient restriction than in other cell types—pointing to the use of these cells as a better means of measuring autophagy in dietary studies.

## 4 Materials and Methods

Details of reagents and software used in this study can be found in Supplementary Table 5.

### 4.1 Human Ethics Approval

The use of the blood samples in the current study was approved by The University of Adelaide Human Research Ethics Committee (Approval HREC H‐2021‐154). Electronic consent for participation was given after participants had been fully informed of the study.

### 4.2 Sample Collection

Blood samples were collected in lithium heparin Vacuette tubes from study participants between April 2023 and August 2024. The study participants include both men and women aged 20 to 50 years who had normal body weight or were overweight (BMI 18.5–29.9 kg/m^2^), recruited from Adelaide, with no history of chronic diseases and without any vaccines or infections within 2 weeks before the visit. The participants were fasted for a minimum of 12 h before blood collection, and the samples were processed within 1 h of collection. Additional information, including sex, ethnicity, weight, and height, was also collected.

### 4.3 Treatment of Whole Blood With CQ

Following collection, 1 mL of whole blood was spilt into two 10 mL conical centrifuge tubes treated with chloroquine diphosphate at a final concentration of 150 μM. The corresponding control tubes were treated with similar amounts of vehicle (water).

### 4.4 PBMC Isolation

PBMCs were isolated using lymphoprep. Blood was mixed with an equal volume of DPBS at a ratio of 1:1 before being carefully layered on top of 15 mL of lymphoprep. PBMCs were collected after being centrifuged for 30 min at 800 x g at room temperature without brake, then washed with DPBS and treated with 1X red blood cell lysis buffer. The PBMCs were then washed twice with DPBS before culturing.

### 4.5 Culture of Isolated PBMCs—Incubation in RPMI Containing 10% FBS or Plasma

In Figures 1 and 5, RPMI containing 10% FBS and diluted cognate plasma (in a 1:1 ratio with DPBS, derived from a lymphoprep‐based PBMC isolation, to achieve similar concentration of PBMCs relative to plasma as in whole blood) was directly compared to whole blood as a medium for the addition of CQ for lysosomal inhibition. To do this, isolated PBMCs were resuspended in either diluted cognate plasma or RPMI containing 10% FBS and incubated with or without CQ or BafA. All samples described here were incubated for 1 h at 37°C with rotation at 10 rpm using a ThermoFisher Scientific Tube Revolver.

### 4.6 Culture of HEK293T Cells

Wild‐type and LC3B knockout HEK293T cells were seeded at 0.5 × 10 ^ 6^6^ cells per well in a 12‐well plate 1 day prior to the experiment in Dulbecco's Modified Eagle's Medium (DMEM) supplemented with 10% FBS. The cells were treated with CQ or its vehicle for 1 h at 37°C before performing flow cytometry.

### 4.7 CRISPR Cas9 Gene Editing (MAP1LC3B KO)

Lentiviral vectors (expressing Cas9 and scramble gRNA 5’‐GTGTAGTTCGACCATTCGTG or gRNA against human LC3B 5’‐CATCCAACCAAAATCCCGGT (pLV[CRISPR]‐hCas9:T2A:Bsd‐U6 > hMAP1LC3B[gRNA#954])) (Vectorbuilder) were transfected into HEK293T cells with plasmids psPAX2 (Addgene #12260) and pCMV‐VSV‐G (Addgene #8454) using lipofection (Lipofectamine 3000, Thermo Fisher, L3000015). Supernatant containing virus 48 h post‐transfection was passed through a 0.45 μM filter. 8 μg/mL polybrene was added to the filtrate. Target cells (HEK293T) were incubated with virus for 24 h and were left to recover for 48 h before selection (6 μg/mL blasticidin, 7d). Monoclonal KO cell lines were generated by sorting cells into a 96‐well plate at 1 cell/well using a BD FACSAria Fusion. Single cell clones were grown for 2–3 weeks before passaging and analysis. Western blotting was used to verify LC3B KO. psPAX2 was a gift from Didier Trono (Addgene plasmid #12260; http://n2t.net/addgene:12260; RRID:Addgene_12260). pCMV‐VSV‐G was a gift from Bob Weinberg (Addgene plasmid #8454; http://n2t.net/addgene:8454; RRID:Addgene_8454).

### 4.8 Culture of Isolated PBMCs—Nutritional Interventions

As in Figures 5 and S5, isolated PBMCs were cultured in various nutritional conditions.

Figure 5: amino acid‐free RPMI prepared according to the manufacturer's instructions containing 10% dFBS, and amino acid‐free RPMI containing 10% dFBS spiked with amino acids, as described in Table S6.

Figure S5 A, B: glucose‐free RPMI containing 10% dFBS and glucose‐free RPMI containing 10% dFBS spiked with glucose at a final concentration of 2 g/L.

CQ and BafA inhibit LC3B‐II degradation by interfering with lysosomal pH [61, 62]. While CQ may induce noncanonical autophagy, BafA has been shown to inhibit this process [60]. Due to the hemolytic effects of the BafA vehicle (DMSO), CQ (which is aqueous) was used for experiments involving whole blood, whereas BafA was used in PBMC culture experiments. We chose to use bafilomycin in PBMC culture experiments because the hemolytic effect of DMSO is less of an issue in the absence of red blood cells, and it is less susceptible to inducing noncanonical autophagy than CQ. To understand which inhibitor has been used, please see the y‐axis of the graph in question. Duplicate isolated PBMCs were treated with Bafilomycin (BafA) at a final concentration of 200 nM, and corresponding control tubes were treated with DMSO as a vehicle control. All cultures were incubated at 37°C for 1 h in 12‐well plates.

### 4.9 Flow Cytometry Staining

Whole blood incubated with CQ and then lysed with red blood cell lysing buffer for 15 min at room temperature (RT); PBMCs after being cultured under different conditions; and wild‐type and LC3B knockout HEK293T cells after being cultured with CQ for 1 h at 37°C; were harvested, washed twice with DPBS, and incubated for 10 min at RT with the recommended amount of fixable viability dye. They were then washed with DPBS before staining with saturated concentrations of surface monoclonal antibodies diluted in BD Horizon Brilliant Stain Buffer Plus for 30 min at 4°C (this step was not applied to HEK cells where cell surface markers were not needed). After staining, cells were washed twice with DPBS and permeabilized with 0.05% saponin for 5 min at RT, and then washed with DPBS to remove LC3B‐I from the cytosol, leaving only LC3B‐II attached to the autophagic membranes. The cells were then fixed with 4% v/v neutral formalin for 15 min at RT; washed with DPBS. Finally, samples were incubated with or without anti‐LC3B Alexa Fluor‐647 (for unstained control) or IgG Alexa Fluor‐647 for IgG control (for PBMCs, wild‐type, and LC3B knockout HEK 293 T cells), diluted 1:50 in DPBS containing 2% bovine serum albumin (BSA) and 0.01% saponin for 1 h at 4°C. The samples were then washed twice with DPBS containing 2% BSA and 0.01% saponin before flow cytometry analysis using a BD FACSymphony A5 Cell Analyzer (BD Biosciences) at a fixed speed. The BD FACSymphony is calibrated daily with BD Cytometer Setup and Tracking Beads.

Surface monoclonal antibodies conjugated to different fluorochromes, including BUV395, BUV615, BUV496, BUV805, BV421, BV480, BV786, BV750, FITC, BB700, PE, PE‐CF594, PE‐Cy7, Live/dead 780, and Alexa647 were employed in this study to characterize different PBMC subpopulations (detailed in Table S5).

### 4.10 Enzyme‐Linked Immunosorbent Assay

ELISA for measuring IL‐1 and IL‐6 was performed according to the manufacturer's protocol. Plasma samples were diluted 1:2 in sample diluent, and standards were prepared as instructed. Then, 50 μL of the diluted samples and standards were added to duplicate wells. Next, 50 μL of the antibody cocktail was added, and the plates were incubated at room temperature on a plate shaker set to 400 rpm for 1 h for IL‐6 samples and 2 h for IL‐1 samples. After incubation, the plates were washed three times with wash buffer and incubated with 100 μL of substrate for 15 min in the dark. The reaction was then stopped, and absorbance was measured at 450 nm.

### 4.11 Quantification and Statistical Analysis

Flow cytometry data analysis was performed using Flowjo 10.8.0 (BD Biosciences, San Jose, CA, USA) for Windows with a gating strategy performed by a single operator (LVPD) with the gating strategy available in Supplementary Figure 1 (D–U). Autophagic flux was quantified as the difference in Geometric Mean Fluorescence Intensity (MFI) of LC3B‐II for each cell population with or without a saturated concentration of CQ or BafA as described by Bensalem et al. [26]:

ΔMFI (LC3B‐II) = (MFI of LC3B‐II with CQ) – (MFI of LC3B‐II without CQ).

ΔMFI (LC3B‐II) = (MFI of LC3B‐II with BafA) – (MFI of LC3B‐II without BafA).

Graphs were generated using GRAPHPAD PRISM version 10.3.0, for Windows (GraphPad Software, La Jolla, CA, USA) or R version 4.3.2 (R Foundation for Statistical Computing, Vienna, Austria)/RStudio (Integrated Development for R. RStudio, PBC, Boston, MA, USA). Data were analyzed using statistical tests described in figure legends (Friedman test, paired Wilcoxon matched‐paired signed rank test, Mann–Whitney test, Spearman correlation, and simple linear regression. All tests were performed as two‐tailed tests, and significant levels are presented as *p < 0.05, **p < 0.01, or ***p < 0.001.

## Author Contributions

L.V.P.D. and T.J.S. designed all the experiments. L.V.P.D. performed all the experiments. L.V.P.D. performed all the data analysis. J.M.C. designed amino acid supplementation. S.S. made LC3B KO cells. A.M. aided in sample collection and processing. J.G. coordinated the parent human study and human data collection. L.V.P.D. and T.J.S. wrote the manuscript. All the authors reviewed the manuscript and provided approval for submission.

## Conflicts of Interest

SAHMRI has patent applications related to this work Australia (Provisional) 2019903 187; 2024900 604; 2019904 822; PCT/AU/2020/050908; United Kingdom GB2204321.0; USA 17/637494.

## Supporting information


Data S1.

## Data Availability

Privacy/ethical restrictions.
