# Supplementary material for: Cell‐Type‐Specific Autophagy in Human Leukocytes
Source: FASEB J. 2025 Jun 12;39(12):e70708. doi: 10.1096/fj.202402377R (PMC12159969; doi:10.1096/fj.202402377R)
Supplement: Supplementary file 1 — Data S1. [file FSB2-39-e70708-s001.pdf]

**Supplementary material**

# Cell-type specific autophagy in human leukocytes

Linh VP Dang[a,b], Alexis Martin[a], Julian M Carosi[a,c], Jemima Gore[d], Sanjna Singh[a], Timothy J Sargeant[a,b]

Affiliations

[a]Lysosomal Health in Ageing, Lifelong Health, South Australian Health and Medical Research Institute (SAHMRI), Adelaide, SA 5000, Australia.

[b]Adelaide Medical School, The University of Adelaide, Adelaide, SA 5000, Australia.

[c]School of Biological Sciences, Faculty of Sciences, Engineering and Technology, The University of Adelaide, Adelaide, SA 5000, Australia.

[d]SAHMRI Clinical Trials Platform (CTP), South Australian Health and Medical Research Institute (SAHMRI), Adelaide, SA 5000, Australia.

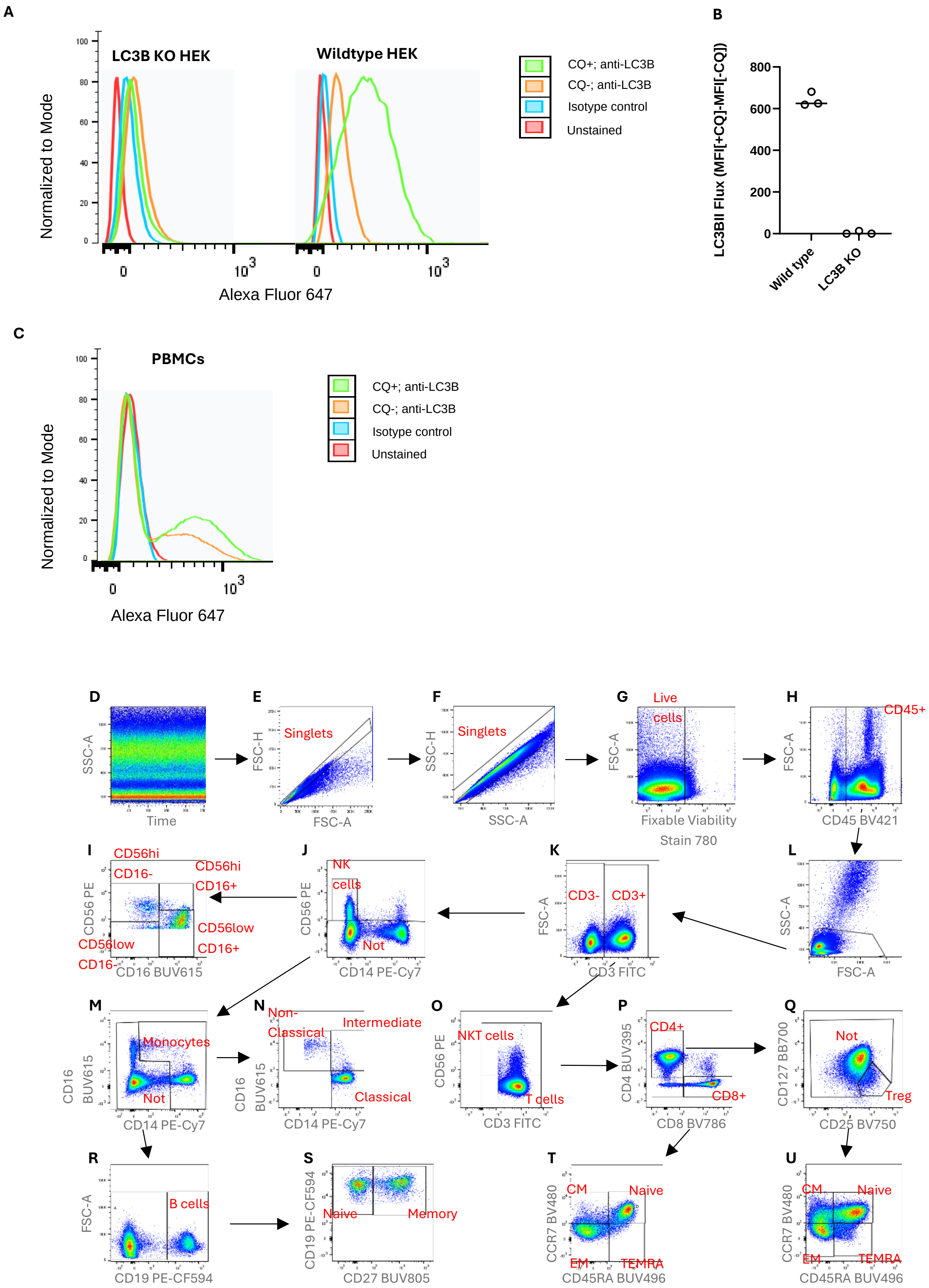
A
LC3B KO HEK
Wildtype HEK
Normalized to Mode
Alexa Fluor 647
CQ+; anti-LC3B
CQ-; anti-LC3B
Isotype control
Unstained
B
LC3BII Flux (MFI[+CQ]-MFI[-CQ])
Wild type
LC3B KO
C
PBMCs
Normalized to Mode
Alexa Fluor 647
CQ+; anti-LC3B
CQ-; anti-LC3B
Isotype control
Unstained
D
SSC-A
Time
E
FSC-H
Singlets
FSC-A
F
SSC-H
Singlets
SSC-A
G
Live cells
FSC-A
Fixable Viability Stain 780
H
CD45+
FSC-A
CD45 BV421
I
CD56hi CD16-
CD56hi CD16+
CD56low CD16+
CD56low CD16-
CD56 PE
CD16 BUV615
J
NK cells
Not
CD56 PE
CD14 PE-Cy7
K
CD3-
CD3+
FSC-A
CD3 FITC
L
SSC-A
FSC-A
M
Monocytes
Not
CD16 BUV615
CD14 PE-Cy7
N
Non-Classical
Intermediate
Classical
CD16 BUV615
CD14 PE-Cy7
O
NKT cells
T cells
CD56 PE
CD3 FITC
P
CD4+
CD8+
CD4 BUV395
CD8 BV786
Q
Not
Treg
CD127 BB700
CD25 BV750
R
B cells
FSC-A
CD19 PE-CF594
S
Naive
Memory
CD19 PE-CF594
CD27 BUV805
T
CM
Naive
EM
TEMRA
CCR7 BV480
CD45RA BUV496
U
CM
Naive
EM
TEMRA
CCR7 BV480
CD45RA BUV496

***Figure S1.***

Flow cytometry staining of wild type and LC3B knockout HEK 293T cells, treated with or without CQ, with unstained and IgG Alexa 647 conditions as controls (A). The plot represents LC3B-II flux from three independent experiments (B). A histogram represents LC3B-II flux in total PBMCs (C).

Gating strategy for the identification of leukocyte populations in participant whole blood samples by flow cytometry: (D-U)

1. Leukocytes were identified by gating on time (D), single cells (E, F), live cells based on low fluorescence of BD Horizon Fixable viability Stain 780 (G), selection of $CD45^+$ cells (H), followed by FSC and SSC gate for monocytes and lymphocytes (L).
2. T cells were identified by staining for CD3 (K), which were further categorized into Natural Killer T (NKT) cells ($CD3^+CD56^+$) and other conventional T cells ($CD3^+CD56^-$) (O). Other conventional T cells were stratified into CD4 and CD8 T cells (P). CD4 T cells ($CD3^+CD4^+$) were then classified into the following sub-populations: Treg ($CD25^+CD127^{low/-}$) (Q) and subsequently divided into naïve ($CD45RA^+CCR7^+$), central memory ($CD45RA^-CCR7^+$), effector memory ($CD45RA^-CCR7^+$), terminally differentiated effector memory (TEMRA; $CD45RA^+CCR7^-$) (U). A similar gating strategy was applied to CD8 T cell sub-populations, including naïve ($CD45RA^+CCR7^+$), central memory ($CD45RA^-CCR7^+$), effector memory ($CD45RA^-CCR7^+$), TEMRA ($CD45RA^+CCR7^-$) (T) based on CD45RA and CCR7 expression.
3. NK cells were identified by gating for $CD3^-CD56^+$ (J), which were then further categorized into $CD56^{hi}CD16^-$, $CD56^{hi}CD16^+$, $CD56^{dim}CD16^-$ and $CD56^{dim}CD16^+$ sub-populations (I).
4. Monocytes were gated based on $CD3^-CD56^-CD14^+$ (M), which were then divided into classical monocytes ($CD14^+CD16^-$), intermediate monocytes ($CD14^+CD16^+$), and non-classical monocytes ($CD14^{low}CD16^+$) (N).
5. B cells were identified by $CD3^-CD56^-CD14^-CD19^+$ (R), which were subsequently characterized into naïve ($CD19^+CD27^-$) and memory B cells ($CD19^+CD27^+$) (S).

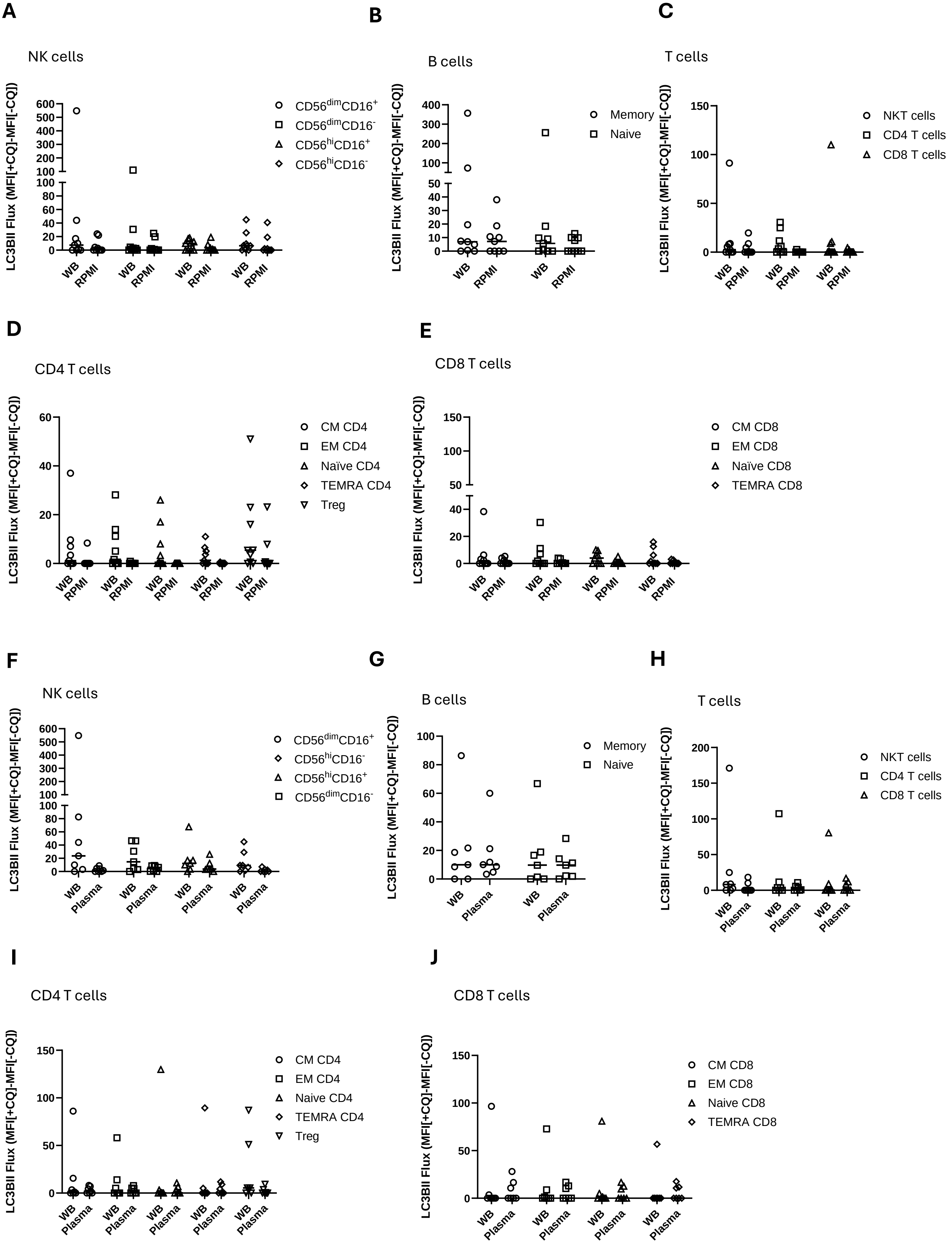
A
NK cells
LC3BII Flux (MFI[+CQ]-MFI[-CQ])
600
500
400
300
200
100
100
80
60
40
20
0
CD56dimCD16+
CD56dimCD16-
CD56hiCD16+
CD56hiCD16-
WB
RPMI
B
B cells
LC3BII Flux (MFI[+CQ]-MFI[-CQ])
Memory
Naive
WB
RPMI
C
T cells
LC3BII Flux (MFI[+CQ]-MFI[-CQ])
NKT cells
CD4 T cells
CD8 T cells
WB
RPMI
D
CD4 T cells
LC3BII Flux (MFI[+CQ]-MFI[-CQ])
CM CD4
EM CD4
Naïve CD4
TEMRA CD4
Treg
WB
RPMI
E
CD8 T cells
LC3BII Flux (MFI[+CQ]-MFI[-CQ])
CM CD8
EM CD8
Naïve CD8
TEMRA CD8
WB
RPMI
F
NK cells
LC3BII Flux (MFI[+CQ]-MFI[-CQ])
CD56dimCD16+
CD56hiCD16-
CD56hiCD16+
CD56dimCD16-
WB
Plasma
G
B cells
LC3BII Flux (MFI[+CQ]-MFI[-CQ])
Memory
Naive
WB
Plasma
H
T cells
LC3BII Flux (MFI[+CQ]-MFI[-CQ])
NKT cells
CD4 T cells
CD8 T cells
WB
Plasma
I
CD4 T cells
LC3BII Flux (MFI[+CQ]-MFI[-CQ])
CM CD4
EM CD4
Naive CD4
TEMRA CD4
Treg
WB
Plasma
J
CD8 T cells
LC3BII Flux (MFI[+CQ]-MFI[-CQ])
CM CD8
EM CD8
Naive CD8
TEMRA CD8
WB
Plasma

***Figure S2***. Comparison of LC3B-II flux in different populations in whole blood (WB) and isolated PBMCs cultured in RPMI medium containing 10% FBS and presented as subpopulations of the following: NK cells (A), B cells (B), T cells (C), CD4 T cells (D), CD8 T cells (E). LC3B-II flux in different populations in whole blood and isolated PBMCs cultured in diluted cognate plasma:DPBS (1:1) and presented as subpopulations of the following: NK cells (F), B cells (G), T cells (H), CD4 T cells (I), CD8 T cells (J). Bars = median. Datapoints = participants.

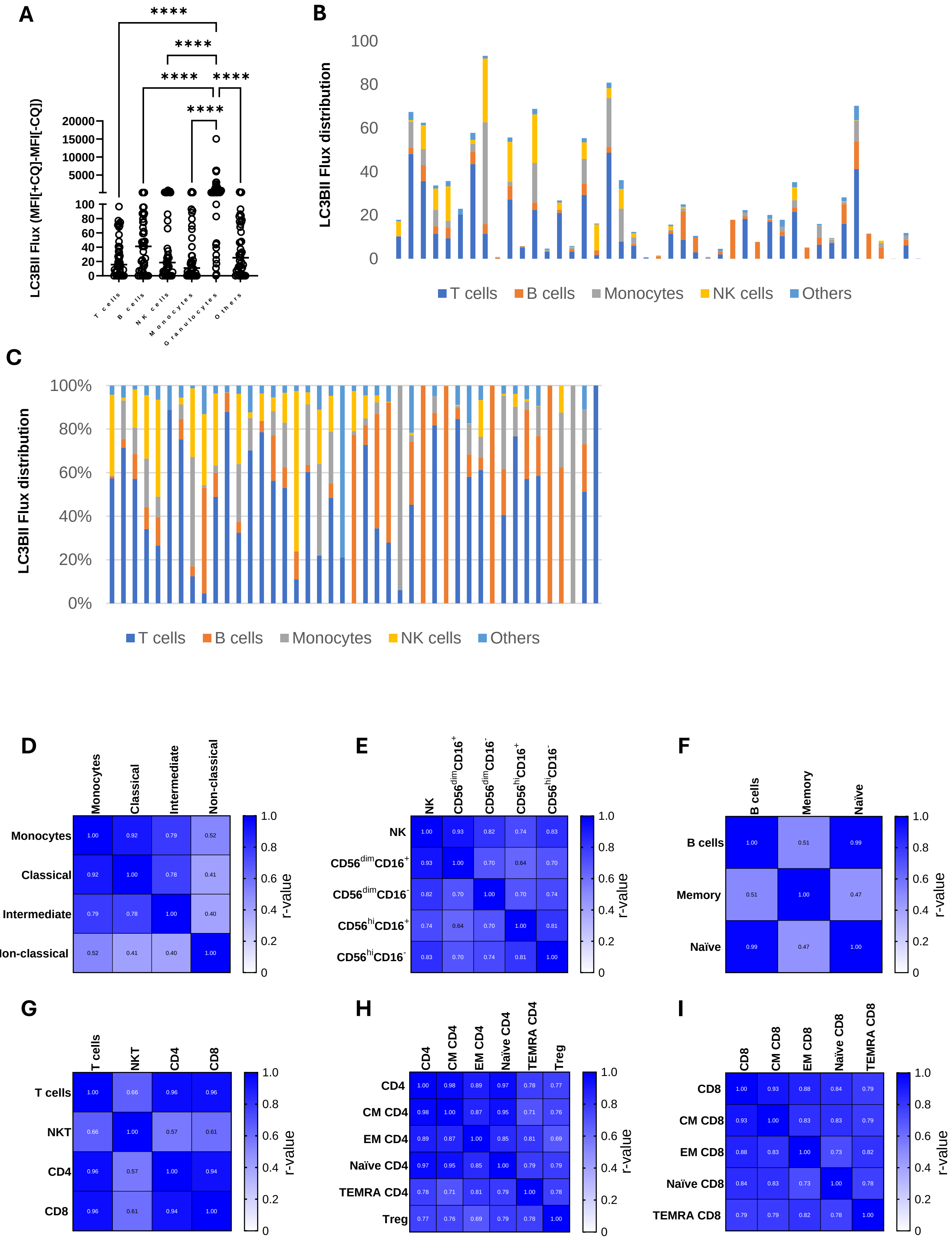

A
LC3BII Flux (MFI[+CQ]-MFI[-CQ])
****
****
****
****
****
20000
15000
10000
5000
100
80
60
40
20
0
T cells
B cells
NK cells
Monocytes
Granulocytes
Others
B
LC3BII Flux distribution
100
80
60
40
20
0
T cells
B cells
Monocytes
NK cells
Others
C
LC3BII Flux distribution
100%
80%
60%
40%
20%
0%
T cells
B cells
Monocytes
NK cells
Others
D
Monocytes
Classical
Intermediate
Non-classical
Monocytes 1.00 0.92 0.79 0.52
Classical 0.92 1.00 0.78 0.41
Intermediate 0.79 0.78 1.00 0.40
Non-classical 0.52 0.41 0.40 1.00
r-value
1.0
0.8
0.6
0.4
0.2
0
E
NK
CD56dimCD16+
CD56dimCD16-
CD56hiCD16+
CD56hiCD16-
NK 1.00 0.93 0.82 0.74 0.83
CD56dimCD16+ 0.93 1.00 0.70 0.64 0.70
CD56dimCD16- 0.82 0.70 1.00 0.70 0.74
CD56hiCD16+ 0.74 0.64 0.70 1.00 0.81
CD56hiCD16- 0.83 0.70 0.74 0.81 1.00
r-value
F
B cells
Memory
Naïve
B cells 1.00 0.51 0.99
Memory 0.51 1.00 0.47
Naïve 0.99 0.47 1.00
r-value
G
T cells
NKT
CD4
CD8
T cells 1.00 0.66 0.96 0.96
NKT 0.66 1.00 0.57 0.61
CD4 0.96 0.57 1.00 0.94
CD8 0.96 0.61 0.94 1.00
r-value
H
CD4
CM CD4
EM CD4
Naïve CD4
TEMRA CD4
Treg
CD4 1.00 0.98 0.89 0.97 0.78 0.77
CM CD4 0.98 1.00 0.87 0.95 0.71 0.76
EM CD4 0.89 0.87 1.00 0.85 0.81 0.69
Naïve CD4 0.97 0.95 0.85 1.00 0.79 0.79
TEMRA CD4 0.78 0.71 0.81 0.79 1.00 0.78
Treg 0.77 0.76 0.69 0.79 0.78 1.00
r-value
I
CD8
CM CD8
EM CD8
Naïve CD8
TEMRA CD8
CD8 1.00 0.93 0.88 0.84 0.79
CM CD8 0.93 1.00 0.83 0.83 0.79
EM CD8 0.88 0.83 1.00 0.73 0.82
Naïve CD8 0.84 0.83 0.73 1.00 0.78
TEMRA CD8 0.79 0.79 0.82 0.78 1.00
r-value

***Figure S3.*** Plot representing LC3B-II flux of different cell populations including T cells, B cells, NK cells, monocytes, granulocytes and others (A). Bars = median. Datapoints = participants. Distribution of LC3B-II flux of each population contributing to the total LC3B-II flux of the PBMC pool (excluding granulocytes): absolute number (B) and relative percentage (C). Heatmap representing the correlation matrix of individual cell populations and its corresponding subpopulations, including monocytes (D), NK cells (E), B cells (F), T cells (G), CD4 T cells (H), and CD8 T cells (I).

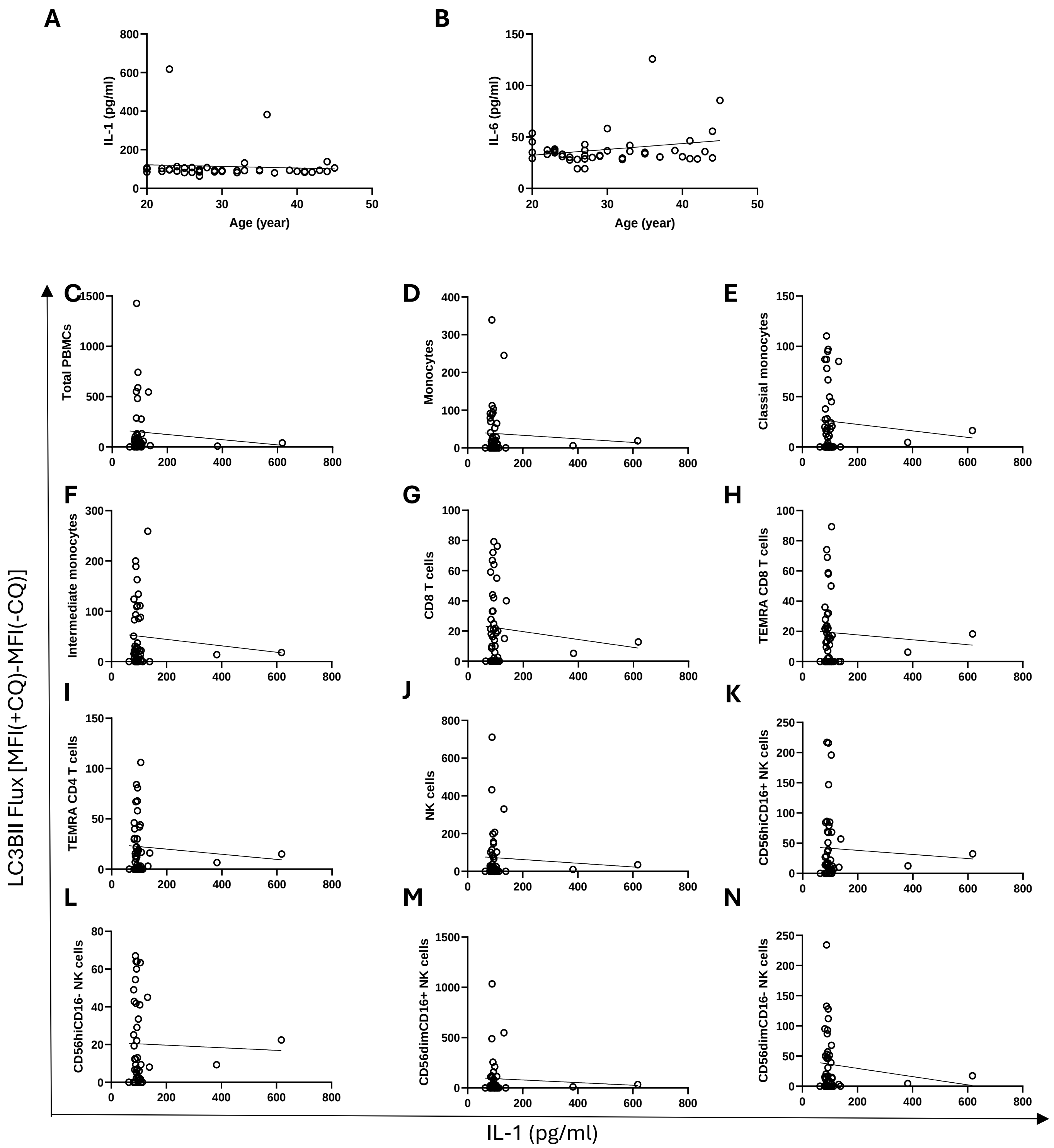
A
IL-1 (pg/ml)
Age (year)
B
IL-6 (pg/ml)
Age (year)
C
Total PBMCs
D
Monocytes
E
Classial monocytes
F
Intermediate monocytes
G
CD8 T cells
H
TEMRA CD8 T cells
I
TEMRA CD4 T cells
J
NK cells
K
CD56hiCD16+ NK cells
L
CD56hiCD16- NK cells
M
CD56dimCD16+ NK cells
N
CD56dimCD16- NK cells
LC3BII Flux [MFI(+CQ)-MFI(-CQ)]
IL-1 (pg/ml)

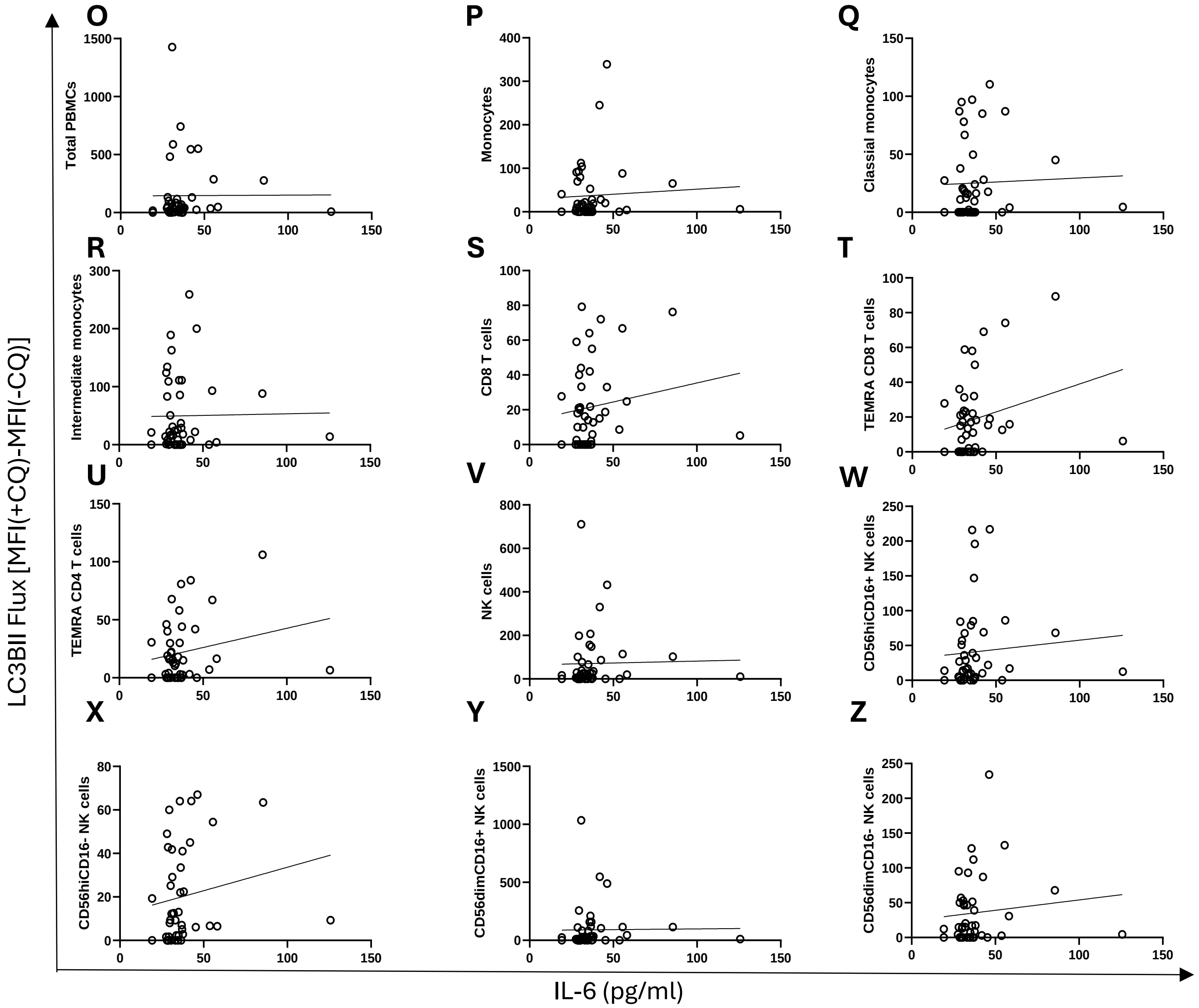


***Figure S4.*** Linear regression analysis of age with IL-1 levels (A) and IL-6 levels (B). Linear regression analysis between IL-1 levels and flux of total PBMCs (C), Monocytes (D), classical monocytes (E), intermediate monocytes (F); CD8 T cells (G), TEMRA CD8 T cells (H) and TEMRA CD4 T cells (I); NK cells (J) $CD56^{hi}CD16^{+}$ NK cells (K), $CD56^{hi}CD16^{-}$ NK cells (L), $CD56^{dim}CD16^{+}$ NK cells (M), and $CD56^{dim}CD16^{-}$ NK cells (N).

Linear regression analysis between IL-6 levels and flux of total PBMCs (O), Monocytes (P), classical monocytes (Q), intermediate monocytes (R); CD8 T cells (S), TEMRA CD8 T cells (T) and TEMRA CD4 T cells (U); NK cells (V) $CD56^{hi}CD16^{+}$ NK cells (W), $CD56^{hi}CD16^{-}$ NK cells (X), $CD56^{dim}CD16^{+}$ NK cells (Y), and $CD56^{dim}CD16^{-}$ NK cells (Z). Datapoints = participants.

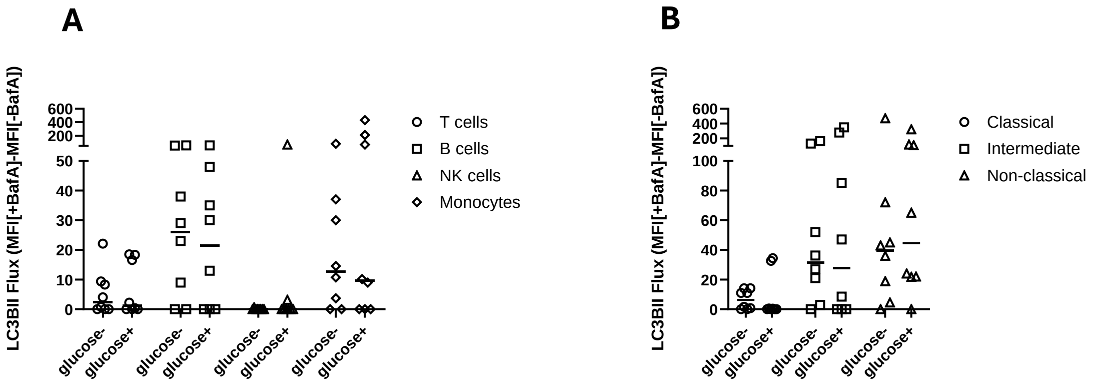


***Figure S5.*** Analysis of LC3B-II flux in different PBMC cell types cultured with glucose-free RPMI containing 10% dFCS (glucose-) or the same medium spiked with glucose (glucose+) (N = 8) and presented as follows: T cells, B cells, NK cells, and monocytes (A), and monocyte sub-populations (B). Bars = median. Datapoints = participants.

## Tables

| **Peripheral blood mononuclear cell type analysed in this study** | **Function** | **Notable roles in ageing or disease** |
|---|---|---|
| **T lymphocytes** (1-3) | • Involved in cell-mediated immune response<br>• Defend the body against infections and cancer<br>• CD4: helper T cells, signalling and activating other immune cells, such as B cells and cytotoxic T cells<br>• CD8: cytotoxic T cells, directly killing infected or cancerous cells | • Age is associated with the remodeling of T cell immunity, which contributes to poor clinical outcomes in age-related diseases such as cancer<br>• Age-related alterations in host and cellular metabolism critically impact the development, maintenance, and function of T cells |
| $CD4^+$ T cells – Central Memory (CM) | • Reside primarily in secondary lymphoid organs<br>• Maintain long-term immunity<br>• Proliferate upon reactivation<br>• Ability to self-renew<br>• Recruit and activate other cells | • Frequency increases with age |
| $CD4^+$ T cells – Effector Memory (EM) | • Found in peripheral tissues and circulation<br>• Quickly respond to previously encountered antigens<br>• Produce cytokines, help B cells, and directly kill infected cells | • Frequency increases with age |
| $CD4^+$ T cells – Naïve | • Respond to new antigens | • The number and function decline with aging, leading to a weakened immune response and increased vulnerability to diseases |
| $CD4^+$ T cells – T effector memory cells re-expressing CD45RA (TEMRA) | • Decline in proliferation potential compared to other memory T cells but strong cytotoxicity and proinflammatory activity<br>• Generate effector molecules including perforins, granzymes, IFN-γ, and TNF-α<br>• High levels of DNA damage and the loss of telomerase activity | • The proportion progressively increases with age |
| $CD4^+$ T cells – Regulatory T cell (Treg) | • Maintain immune homeostasis by suppressing excessive immune responses and preventing autoimmune diseases<br>• Control the activation and activity of other immune cells, including CD4 helper T cells, cytotoxic CD8 T cells, and B cells | • The number tends to increase with age |
| $CD8^+$ T cells – Central Memory (CM) | • Maintain long-term immune memory and exhibit high proliferative potential upon reactivation<br>• Produce cytokines like IL-2 to enhance immune responses after activation | • Frequency tends to increase slightly or remain stable with age |

| | | |
|---|---|---|
| $CD8^+$ T cells – Effector Memory (EM) | • Provide rapid effector responses upon antigen re-exposure<br>• Rapidly produce cytokines such as IFN-γ and TNF-α | • Frequency increases significantly with age |
| $CD8^+$ T cells – Naïve | • Play a crucial role in clearing intracellular pathogens and tumor cells by differentiating into effector and memory cells upon antigen recognition | • Frequency decreases with age |
| $CD8^+$ T cells – T effector memory cells re-expressing CD45RA (TEMRA) | • Play a crucial role in both immune responses and immune senescence<br>• High level of cytotoxicity, rapid effector function, and ability to accumulate with age or chronic antigen stimulation<br>• Contribute to antiviral responses, immune senescence, chronic inflammation, and potentially in graft rejection | • Frequency increases with age |
| **B lymphocytes (4)** | • Play a crucial role in the immune system by producing antibodies to combat infections | • Undergo significant changes with age affecting function and contributing to the age-related decline in immune response<br>• Decrease in the overall number and impairment in antibody production with age, leading to increased risk of infections, autoimmune diseases, and reduced vaccine efficacy in elderly |
| Naïve B lymphocytes | • B cells that have not yet encountered their specific antigen<br>• Respond slowly upon encountering an antigen (primary immune response) | • The percentages and numbers are significantly decreased with age |
| Memory B lymphocytes | • Crucial for long-lasting immunity after an initial infection or vaccination<br>• Play a key role in the secondary immune response by rapidly recognizing and responding to previously encountered antigens by differentiating into long-lived plasma cells, producing high-affinity antibodies that protect against subsequent infections | • The proportion generally increases with age |
| **Natural Killer Cells (5-8)** | • Belong to innate immune response<br>• Crucial for detecting and eliminating infected, stressed, or cancerous cells<br>• Act as a first line of defense against various pathogens and tumors | • Play a significant role in aging, exhibit functional changes that impact the immune system and overall health<br>• Decline activity and function with age, leading to increased susceptibility to infections, inflammation, and potentially contributing to the development of age-related diseases |
| $CD56^{dim}CD16^+$ Natural Killer Cells | • Mediate natural and antibody-dependent cellular cytotoxicity, | • Frequency increases with age or unchanged |

| | | |
|---|---|---|
| | exhibiting high levels of perforin and enhanced killing | |
| $CD56^{dim}CD16^{-}$ Natural Killer Cells | • The role of this population is generally unknown, they are suggested to play a role in cancer since they are often found within the tumor microenvironment | • Frequency increases with age |
| $CD56^{hi}CD16^{+}$ Natural Killer Cells | • Have strong cell-mediated cytotoxicity as well as antibody-dependent cell-mediated cytotoxicity<br>• Low cytokine production capacity | • Frequency decreases with age |
| $CD56^{hi}CD16^{-}$ Natural Killer cells | • Produce cytokines, predominantly IFN-γ, thus linking innate and adaptive immune system | • Frequency increases with age |
| **Monocytes(1, 9, 10)** | • Crucial for the body's defense against infection and inflammation<br>• Circulate in the bloodstream and migrate to tissues, where they differentiate into macrophages or dendritic cells, playing essential roles in immunity and tissue repair | • The number increases with age<br>• Play a significant role in the aging process, particularly in the context of inflammaging<br>• Undergo functional and phenotypic changes, leading to increased pro-inflammatory cytokine production and contributing to chronic low-grade inflammation, resulting in increased susceptibility to infections and impact various age-related diseases |
| Classical monocytes | • The first responders to infection and inflammation<br>• Phagocytic<br>• Produce IL-6, TNF-α, IL-1β (pro-inflammatory)<br>• Actively recruited to inflamed or infected tissues<br>• Efficient phagocytosis and reactive oxygen species (ROS) production | • The frequency decreases with age<br>• The functions become substantially dysregulated with advanced age |
| Intermediate monocytes | • Act as a bridge between innate and adaptive immunity<br>• Produce pro-inflammatory cytokines and generate ROS<br>• Present antigens to T cells | • The frequency increases with age |
| Non-classical monocyte | • Patrol endothelium, scavenge debris<br>• Surveil and repair tissue<br>• Produce anti-inflammatory cytokines like IL-10 | • The frequency increases with age |

***Table S1***. PBMC cell types investigated in this study and their functions.

| | | Total (n=43) | Males (n=19) | Female (n=24) | p value |
|---|---|---|---|---|---|
| ***Age (years)*** | | 28 (20-45) | 27(20-44) | 29.5 (20-45) | 0.19 |
| ***Gender, female n (%)*** | | 24 (55.8%) | | | |
| ***Body mass index (BMI, kg/m²)*** | | 23.8 (19.1-30.9) | 25.1 (20.4-30.9) | 21.9 (19.1-29.5) | 0.05 |
| ***Ethnicity*** | | | | | |
| | Caucasian | 18(41.6%) | 6 (31.6%) | 12(50%) | 0.14 |
| | Asian | 24 (58.1%) | 13 (68.4%) | 11(45.8%) | |
| | Others | 1(2.3%) | 0 | 1(4.2%) | |

***Table S2.*** Participant characteristics, analyzed by Mann-Whitney and Kruskal Wallis test.

| **r/p value** | ***Total*** | ***T cells*** | ***B cells*** | ***Monocytes*** | ***NK cells*** |
|---|---|---|---|---|---|
| ***Total*** | | **<0.01** | **<0.01** | **<0.01** | **<0.01** |
| ***T cells*** | 0.63 | | **0.05** | **<0.01** | **<0.01** |
| ***B cells*** | 0.51 | 0.31 | | 0.10 | **0.01** |
| ***Monocytes*** | 0.61 | 0.74 | 0.25 | | **<0.01** |
| ***NK cells*** | 0.69 | 0.63 | 0.38 | 0.72 | |

Table S3A.

| ***r/p value*** | ***T cells*** | ***NKT*** | ***CD4*** | ***CD8*** |
|---|---|---|---|---|
| ***T cells*** | | **<0.01** | **<0.01** | **<0.01** |
| ***NKT*** | 0.66 | | **<0.01** | **<0.01** |
| ***CD4*** | 0.96 | 0.57 | | **<0.01** |
| ***CD8*** | 0.96 | 0.61 | 0.94 | |

Table S3B.

| ***r/p value*** | ***CD4*** | ***CM*** | ***EM*** | ***Naïve*** | ***TEMRA*** | ***Treg*** |
|---|---|---|---|---|---|---|
| ***CD4*** | | **<0.01** | **<0.01** | **<0.01** | **<0.01** | **<0.01** |
| ***CM*** | 0.976 | | **<0.01** | **<0.01** | **<0.01** | **<0.01** |
| ***EM*** | 0.894 | 0.871 | | **<0.01** | **<0.01** | **<0.01** |
| ***Naïve*** | 0.967 | 0.947 | 0.850 | | **<0.01** | **<0.01** |
| ***TEMRA*** | 0.783 | 0.708 | 0.809 | 0.793 | | **<0.01** |
| ***Treg*** | 0.771 | 0.759 | 0.694 | 0.788 | 0.778 | |

Table S3C.

| ***r/p value*** | ***CD8*** | ***CM*** | ***EM*** | ***Naïve*** | ***TEMRA*** |
|---|---|---|---|---|---|
| ***CD8*** | | **<0.01** | **<0.01** | **<0.01** | **<0.01** |
| ***CM*** | 0.928 | | **<0.01** | **<0.01** | **<0.01** |
| ***EM*** | 0.877 | 0.831 | | **<0.01** | **<0.01** |
| ***Naïve*** | 0.844 | 0.834 | 0.732 | | **<0.01** |
| ***TEMRA*** | 0.790 | 0.790 | 0.823 | 0.775 | |

Table S3D.

| ***r/p value*** | ***B cells*** | ***Memory*** | ***Naïve*** |
|---|---|---|---|
| ***B cells*** | | **<0.01** | **<0.01** |
| ***Memory*** | 0.511 | | **<0.01** |
| ***Naïve*** | 0.987 | 0.473 | |

Table S3E.

| ***r/p value*** | ***Monocytes*** | ***Classical*** | ***Intermediate*** | ***Non-classical*** |
|---|---|---|---|---|
| ***Monocytes*** | | **<0.01** | **<0.01** | **<0.01** |
| ***Classical*** | 0.915 | | **<0.01** | **<0.01** |
| ***Intermediate*** | 0.793 | 0.775 | | **<0.01** |
| ***Non-classical*** | 0.523 | 0.414 | 0.403 | |

Table S3F.

| ***r/p value*** | ***NK cells*** | ***$CD56^{dim}CD16^{+}$*** | ***$CD56^{dim}CD16^{-}$*** | ***$CD56^{hi}CD16^{+}$*** | ***$CD56^{hi}CD16^{-}$*** |
|---|---|---|---|---|---|
| ***NK cells*** | | **<0.01** | **<0.01** | **<0.01** | **<0.01** |
| ***$CD56^{dim}CD16^{+}$*** | 0.932 | | **<0.01** | **<0.01** | **<0.01** |
| ***$CD56^{dim}CD16^{-}$*** | 0.820 | 0.704 | | **<0.01** | **<0.01** |
| ***$CD56^{hi}CD16^{+}$*** | 0.739 | 0.636 | 0.704 | | **<0.01** |
| ***$CD56^{hi}CD16^{-}$*** | 0.835 | 0.700 | 0.743 | 0.814 | |

Table S3G.

| r/p value | Total | NK T | CM CD4 | EM CD4 | Naïve CD4 | TEMRA CD4 | Treg | CM CD8 | EM CD8 | Naïve CD8 | TEMRA CD8 | Memory B | Naïve B | CM | IM | NM | NK1 | NK2 | NK3 | NK4 |
|---|---|---|---|---|---|---|---|---|---|---|---|---|---|---|---|---|---|---|---|---|
| Total | | **<0.01** | **<0.01** | **0.013** | **<0.01** | **0.018** | **<0.01** | **<0.01** | **<0.01** | **<0.01** | **<0.01** | **<0.01** | **<0.01** | **<0.01** | **0.012** | **<0.01** | **<0.01** | **<0.01** | **0.002** | **<0.01** |
| NK T | 0.487 | | | **<0.01** | **<0.01** | **0.011** | **<0.01** | **<0.01** | **<0.01** | **<0.01** | **<0.01** | **<0.01** | **<0.01** | **<0.01** | **0.029** | **0.020** | **0.003** | **0.002** | **0.002** | **<0.01** |
| CM CD4 | 0.576 | 0.579 | | **<0.01** | **<0.01** | **<0.01** | **<0.01** | **<0.01** | **<0.01** | **<0.01** | **<0.01** | **<0.01** | **0.022** | **<0.01** | **<0.01** | **0.040** | **0.005** | **<0.01** | **<0.01** | **<0.01** |
| EM CD4 | 0.376 | 0.479 | 0.871 | | **<0.01** | **<0.01** | **<0.01** | **<0.01** | **<0.01** | **<0.01** | **<0.01** | **<0.01** | 0.189 | **<0.01** | **0.007** | 0.347 | **0.040** | 0.003 | **<0.01** | **<0.01** |
| Naïve CD4 | 0.602 | 0.604 | 0.947 | 0.850 | | **<0.01** | **<0.01** | **<0.01** | **<0.01** | **<0.01** | **<0.01** | **<0.01** | **<0.01** | **<0.01** | **<0.01** | **0.032** | **0.001** | **<0.01** | **<0.01** | **<0.01** |
| TEMRA CD4 | 0.358 | 0.383 | 0.708 | 0.809 | 0.793 | | **<0.01** | **<0.01** | **<0.01** | **<0.01** | **<0.01** | **<0.01** | 0.102 | **<0.01** | **0.008** | 0.256 | **0.011** | **<0.01** | **<0.01** | **<0.01** |
| Treg | 0.452 | 0.486 | 0.759 | 0.694 | 0.788 | 0.778 | | **<0.01** | **<0.01** | **<0.01** | **<0.01** | **<0.01** | **0.012** | **<0.01** | **0.014** | 0.289 | **0.002** | **<0.01** | **<0.01** | **<0.01** |
| CM CD8 | 0.512 | 0.569 | 0.890 | 0.775 | 0.866 | 0.695 | 0.736 | | **<0.01** | **<0.01** | **<0.01** | **<0.01** | **0.009** | **<0.01** | **<0.01** | 0.091 | **0.004** | **<0.01** | **<0.01** | **<0.01** |
| EM CD8 | 0.466 | 0.494 | 0.813 | 0.872 | 0.805 | 0.824 | 0.649 | 0.831 | | **<0.01** | **<0.01** | **<0.01** | **0.035** | **<0.01** | **<0.01** | 0.156 | **0.002** | **<0.01** | **<0.01** | **<0.01** |
| Naïve CD8 | 0.531 | 0.510 | 0.864 | 0.800 | 0.875 | 0.730 | 0.798 | 0.834 | 0.732 | | **<0.01** | **<0.01** | **0.023** | **<0.01** | **0.005** | **0.019** | **0.004** | **<0.01** | **<0.01** | **<0.01** |
| TEMRA CD8 | 0.471 | 0.466 | 0.741 | 0.737 | 0.828 | 0.883 | 0.859 | 0.790 | 0.823 | 0.775 | | **<0.01** | **<0.01** | **<0.01** | **<0.01** | 0.324 | **<0.01** | **<0.01** | **<0.01** | **<0.01** |
| Memory B | 0.546 | 0.494 | 0.651 | 0.583 | 0.719 | 0.704 | 0.695 | 0.666 | 0.735 | 0.674 | 0.783 | | **<0.01** | **<0.01** | **<0.01** | **0.004** | **<0.01** | **<0.01** | **<0.01** | **<0.01** |
| Naïve B | 0.480 | 0.436 | 0.349 | 0.204 | 0.439 | 0.253 | 0.379 | 0.393 | 0.323 | 0.346 | 0.456 | 0.473 | | 0.010 | 0.314 | 0.077 | **0.018** | 0.003 | 0.024 | 0.017 |
| CM | 0.624 | 0.511 | 0.742 | 0.627 | 0.759 | 0.512 | 0.630 | 0.653 | 0.674 | 0.688 | 0.646 | 0.716 | 0.387 | | **<0.01** | **0.006** | **<0.01** | **<0.01** | **<0.01** | **<0.01** |
| IM | 0.378 | 0.333 | 0.458 | 0.408 | 0.509 | 0.401 | 0.374 | 0.432 | 0.527 | 0.420 | 0.503 | 0.647 | 0.157 | 0.775 | | **0.007** | **<0.01** | **0.002** | **0.002** | **<0.01** |
| NM | 0.635 | 0.353 | 0.314 | 0.147 | 0.327 | 0.177 | 0.166 | 0.261 | 0.220 | 0.356 | 0.154 | 0.435 | 0.273 | 0.414 | 0.403 | | **0.005** | **0.023** | 0.115 | 0.062 |
| NK1 | 0.604 | 0.447 | 0.419 | 0.314 | 0.472 | 0.385 | 0.463 | 0.433 | 0.465 | 0.432 | 0.532 | 0.610 | 0.359 | 0.611 | 0.531 | 0.423 | | **<0.01** | **<0.01** | **<0.01** |
| NK2 | 0.681 | 0.453 | 0.571 | 0.445 | 0.682 | 0.556 | 0.648 | 0.601 | 0.567 | 0.584 | 0.731 | 0.703 | 0.449 | 0.686 | 0.462 | 0.346 | 0.704 | | **<0.01** | **<0.01** |
| NK3 | 0.461 | 0.468 | 0.613 | 0.549 | 0.656 | 0.643 | 0.676 | 0.634 | 0.628 | 0.584 | 0.706 | 0.611 | 0.343 | 0.581 | 0.464 | 0.244 | 0.636 | 0.704 | | **<0.01** |
| NK4 | 0.588 | 0.556 | 0.797 | 0.699 | 0.793 | 0.629 | 0.740 | 0.740 | 0.732 | 0.739 | 0.743 | 0.682 | 0.363 | 0.836 | 0.593 | 0.287 | 0.700 | 0.743 | 0.814 | |

Table S3H.

***Table S3.*** Correlation of total LC3B-II flux with major populations and subpopulations analyzed by correlation matrix with data presented as Spearman r and p value as follows: Total with major populations (A), T cells (B), CD4 T cells (C), CD8 T cells (D), B cells (E), monocytes (F), NK cells (G), total LC3B-II flux with subpopulations (H). CM (classical monocytes), IM (intermediate monocytes), NM (non-classical monocytes), NK1 ($CD56^{dim}CD16^{+}$ NK cells), NK2 ($CD56^{dim}CD16^{-}$ NK cells), NK3 ($CD56^{hi}CD16^{+}$NK cells), NK4 ($CD56^{hi}CD16^{-}$ NK cells).

| LC3BII flux | | | Total (n=43) | Males (n=19) | Female (n=24) | p value |
|---|---|---|---|---|---|---|
| ***Total*** | | | 41.4 (0.1-1427) | 24.9(0.1-742) | 61.5(0.1-1427) | 0.059 |
| ***T cells*** | | | 15.8 (0-96.65) | 8.4 (0-70) | 21.75 (0-96.65) | 0.059 |
| | *NKT cells* | | 34 (0-571) | 12.8 (0-539) | 40.75 (0-571) | 0.16 |
| | *CD4 T cells* | | 13.9 (0-100.6) | 4.5 (0-61) | 18 (0-100.6) | 0.14 |
| | | Central memory | 14.9 (0-93.2) | 9.4 (0-63) | 17.35 (0-93.2) | 0.14 |
| | | Effector memory | 14 (0-84) | 7.5 (0-79.7) | 16.2 (0-84) | 0.49 |
| | | Naïve | 14.5 (0-113.3) | 7.3 (0-64) | 15.2 (0-113.3) | 0.21 |
| | | TEMRA | 15.11 (0-106) | 13.1 (0-80.8) | 15.56 (0-106) | 0.79 |
| | | Regulatory | 9.3 (0-177.72) | 7.1 (0-56.8) | 12.98 (0-177.72) | 0.49 |
| | *CD8 T cells* | | 15 (0-79.2) | 86. (0-66.8) | 19 (0-79.2) | 0.15 |
| | | Central memory | 14.7 (0-407) | 12.8 (0-61.4) | 16.7 (0-407) | 0.19 |
| | | Effector memory | 11 (0-82.1) | 6.5 (0-75) | 14.73 (0-82.1) | 0.3 |
| | | Naïve | 9.6 (0-135.1) | 3 (0-74) | 15.5 (0-135.1) | 0.057 |
| | | TEMRA | 15 (0-89.4) | 12.6 (0-74.1) | 15.45 (0-89.4) | 0.9 |
| ***B cells*** | | | 41.1 (0-120.1) | 46.9 (0-110) | 32.75 (0-120.1) | 0.98 |
| | *Naïve* | | 36 (0-148) | 47.5 (0-134) | 28.55 (0-148) | 0.79 |
| | *Memory* | | 18.62 (0-126.9) | 7 (0-87.2) | 20.96 (0-126.9) | 0.17 |
| ***Monocytes*** | | | 10.9 (0-339) | 3 (0-93) | 18.39 (0-339) | **0.03** |
| | *Classical monocytes* | | 10.2 (0.79-21.8) | 8.41 (0.79-17.3) | 10.35 (1.67-21.8) | 0.62 |
| | *Intermediate monocytes* | | 21 (0-259) | 21 (0-111) | 20.195 (0-259) | 0.36 |
| | *Non-classical monocytes* | | 78 (0-3175) | 45 (0-174) | 180 (0-3175) | **<0.01** |
| ***NK cells*** | | | 18.49 (0-711) | 11.9 (0-198) | 22.31 (0-711) | 0.23 |
| | $CD56^{dim}$ $CD16^{+}$ | | 23.65 (0-1034) | 24.3 (0-257) | 17.86 (0-1034) | 0.46 |
| | $CD56^{dim}$ $CD16^{-}$ | | 15 (0-234) | 8.8 (0-132.5) | 17.15 (0-234) | 0.21 |
| | $CD56^{hi}$ $CD16^{+}$ | | 15.6 (0-217) | 15.6 (0-216) | 15.57 (0-217) | 0.47 |
| | $CD56^{hi}$ $CD16^{-}$ | | 9.38 (0-67) | 7 (0-64) | 10.79 (0-67) | 0.38 |

***Table S4.*** Sex differences for autophagic flux in different cell populations analyzed by Mann-Whitney test.

| Reagents or Resources | Source | Identifier |
|---|---|---|
| ***Antibodies*** | | |
| BD Horizon BB700 Mouse Anti-Human CD127 | BD Biosciences | 566398<br>Clone HIL-7R-M21 |
| BD Horizon BUV395 Mouse Anti-Human CD4 | BD Biosciences | 563550<br>Clone K3 |
| BD Horizon BUV805 Mouse Anti-Human CD27 | BD Biosciences | 569167<br>Clone L128 |
| BD Horizon BV421 Mouse Anti-Human CD45 | BD Biosciences | 563879<br>Clone HI30 |
| BD Horizon BV480 Rat Anti-Human CCR7 (CD197) | BD Biosciences | 566099<br>Clone 3D12 |
| BD Horizon BV786 Mouse Anti-Human CD8 | BD Biosciences | 563823<br>Clone RPA-T8 |
| BD Horizon Fixable Viability Stain780 | BD Biosciences | 565388 |
| BD Horizon PE-CF594 Mouse Anti-Human CD19 | BD Biosciences | 562294<br>Clone HIB19 |
| BD OptiBuild BUV496 Mouse Anti-Human CD45RA | BD Biosciences | 741182<br>Clone 5H9 |
| BD OptiBuild BUV615 Mouse Anti-Human CD16 | BD Biosciences | 751323<br>Clone B73.1 |
| BD OptiBuild BV750 Mouse Anti-Human CD25 | BD Biosciences | 747290<br>Clone 2A3 |
| BD Pharmingen FITC Mouse Anti-Human CD3 | BD Biosciences | 555339<br>Clone HIT3a |
| BD Pharmingen FITC Mouse Anti-Human CD56 | BD Biosciences | 555516<br>Clone B159 |
| BD Pharmingen PE-Cy7 Mouse Anti-Human CD14 | BD Biosciences | 557742<br>Clone M5E2 |
| LC3B (E5Q2K) Mouse mAb (Alexa Fluor® 647 Conjugate) | Cell Signaling Technology | 18577S |
| ***Chemicals and consumables*** | | |
| Bafilomycin A1 | Selleck Chemicals | S1413 |
| BD Horizon Brilliant Stain Buffer Plus | BD Biosciences | 566385 |
| BD™ Cytometer Setup and Tracking Beads | BD Biosciences | 642412 |
| Bovine serum albumin | Sigma Aldrich | A9647-100G |
| Chloroquine diphosphate | Sigma Aldrich | C6628 |
| Dulbecco's modifed Eagle's medium (DMEM, high glucose, no glutamine) | Thermo Fisher Scientific | 11960044 |
| Dulbecco's phosphate-buffered saline (DPBS) | GIBCO, Thermo Fisher Scientific | 14 190 136 |
| Fetal bovine serum (undialyzed) | Life Technologies | 10 099–141 |
| Fetal bovine serum, dialyzed (d.FBS) | Thermo Fisher Scientific | A3382001 |
| Lithium heparin Vacu3tte tube 9mL | Greiner Bio-One | 455.084 |
| Lymphoprep | StemCell Technologies | 07811 |
| Neutral formalin | Thermo Fisher Scientific | BSPFS426.2.5 |
| Red blood cell lysis buffer | BD Biosciences | 555 899 |

| | | |
|---|---|---|
| RPMI 1640 medium | Life Technologies | R8758 |
| RPMI 1640 medium w/o amino acids, sodium phosphate (powder) | US Biological | R8999-04A |
| RPMI 1640 medium, no glucose | Thermo Fisher Scientific | 11879020 |
| Saponin from quillaja bark | Sigma Aldrich | S4521-10G |
| Glucose solution | Thermo Fisher Scientific | A2494001 |
| Sodium bicarbonate | Sigma Aldrich | S5761 |
| Sodium phosphate dibasic anhydrous | Sigma Aldrich | S9763 |
| L-Aspartic acid | Sigma Aldrich | A9256-100G |
| L-Serine | Sigma Aldrich | S4311-25G |
| L-Threonine | Sigma Aldrich | T8625-10G |
| L-Asparagine | Sigma Aldrich | A0884-25G |
| L-Cysteine | Sigma Aldrich | C7352-25G |
| L-Alanine | Sigma Aldrich | A7469-25G |
| L-Methionine | Sigma Aldrich | M9625-25G |
| L-Phenylalanine | Sigma Aldrich | P2126-100G |
| L-Tyrosine | Sigma Aldrich | T3754-50G |
| L-Tryptophan | Sigma Aldrich | T0254-25G |
| L-Leucine | Sigma Aldrich | L8912 |
| L-Valine | Sigma Aldrich | V0513 |
| L-Isoleucine | Sigma Aldrich | I7403 |
| L-Lysine | Sigma Aldrich | L5501 |
| L-Histidine | Sigma Aldrich | H8000 |
| L-Arginine | Sigma Aldrich | A8094 |
| L-Glutamic acid (monosodium salt hydrate) | Sigma Aldrich | G5889 |
| L-Proline | Sigma Aldrich | P03080 |
| Glycine | Millipore | VP709001610 |
| L-Glutamine | Sigma Aldrich | G7513 |
| Human IL-6 ELISA Kit | Abcam | AB178013 |
| Human IL-1 beta ELISA Kit | Abcam | AB214025 |
| ***Software and Algorithms*** | | |
| Flowjo 10.8.0 for Window | Tree Star | |
| GRAPHPAD PRISM, version 10.1.0 for Windows | GraphPad | |
| R version 4.3.2 | The R Foundation www.r-project.org | |
| RStudio | www.rstudio.com | |

***Table S5***. Reagents used in the study.

| Amino Acids | Molecular Weight | Concentration (mM) |
|---|---|---|
| Glycine | 75 | 0.133 |
| L-Arginine | 174 | 1.149 |
| L-Asparagine | 132 | 0.379 |
| L-Aspartic acid | 133 | 0.15 |
| L-Cystine 2HCl | 313 | 0.208 |
| L-Glutamic Acid | 147 | 0.136 |
| L-Glutamine* | 146 | 2.055 |
| L-Histidine | 155 | 0.097 |
| L-Isoleucine | 131 | 0.382 |
| L-Leucine | 131 | 0.382 |
| L-Lysine hydrochloride | 183 | 0.219 |
| L-Methionine | 149 | 0.101 |
| L-Phenylalanine | 165 | 0.091 |
| L-Proline | 115 | 0.174 |
| L-Serine | 105 | 0.286 |
| L-Threonine | 119 | 0.168 |
| L-Tryptophan | 204 | 0.025 |
| L-Tyrosine disodium salt dihydrate | 261 | 0.111 |
| L-Valine | 117 | 0.171 |
| L-Alanine | 89.09 | 0.4 |

***Table S6***. Amino acids and concentrations spiked into aa$^{+}$ condition in Fig. 5

**Supplementary references**


1. Kverneland, A. H., Streitz, M., Geissler, E., Hutchinson, J., Vogt, K., Boes, D., Niemann, N., Pedersen, A. E., Schlickeiser, S., and Sawitzki, B. (2016) Age and gender leucocytes variances and references values generated using the standardized ONE-Study protocol. *Cytometry A* **89**, 543-564
2. Kaszubowska, L., Foerster, J., and Kmiec, Z. (2022) NKT-like (CD3 + CD56+) cells differ from T cells in expression level of cellular protective proteins and sensitivity to stimulation in the process of ageing. *Immun Ageing* **19**, 18
3. Kumar, B. V., Connors, T. J., and Farber, D. L. (2018) Human T Cell Development, Localization, and Function throughout Life. *Immunity* **48**, 202-213
4. Frasca, D., and Blomberg, B. B. (2011) Aging affects human B cell responses. *J Clin Immunol* **31**, 430-435
5. Giansanti, M., Theinert, T., Boeing, S. K., Haas, D., Schlegel, P. G., Vacca, P., Nazio, F., and Caruana, I. (2023) Exploiting autophagy balance in T and NK cells as a new strategy to implement adoptive cell therapies. *Mol Cancer* **22**, 201
6. Keating, S. E., Zaiatz-Bittencourt, V., Loftus, R. M., Keane, C., Brennan, K., Finlay, D. K., and Gardiner, C. M. (2016) Metabolic Reprogramming Supports IFN-gamma Production by CD56bright NK Cells. *J Immunol* **196**, 2552-2560
7. Poli, A., Michel, T., Theresine, M., Andres, E., Hentges, F., and Zimmer, J. (2009) CD56bright natural killer (NK) cells: an important NK cell subset. *Immunology* **126**, 458-465
8. Vujanovic, L., Chuckran, C., Lin, Y., Ding, F., Sander, C. A., Santos, P. M., Lohr, J., Mashadi-Hossein, A., Warren, S., White, A., Huang, A., Kirkwood, J. M., and Butterfield, L. H. (2019) CD56(dim) CD16(-) Natural Killer Cell Profiling in Melanoma Patients Receiving a Cancer Vaccine and Interferon-alpha. *Front Immunol* **10**, 14
9. Ozanska, A., Szymczak, D., and Rybka, J. (2020) Pattern of human monocyte subpopulations in health and disease. *Scand J Immunol* **92**, e12883
10. Basu, S., Ulbricht, Y., and Rossol, M. (2025) Healthy and premature aging of monocytes and macrophages. *Front Immunol* **16**, 1506165
